# Recent advances in nanomaterial-based strategies for chronic pain alleviation

**DOI:** 10.1016/j.mtbio.2026.103279

**Published:** 2026-05-23

**Authors:** Zihan Xue, Chengfeng Zhang, Jingyi Wang, Jianing Li, Faming Wang, Zhongping Chen, Yan Zhang

**Affiliations:** Institute of Special Environmental Medicine, School of Public Health, Nantong University, Nantong, 226019, China

**Keywords:** Nanomaterials, Chronic pain, Nanocarrier, Nanozyme, Extracellular vesicle

## Abstract

Chronic pain profoundly impacts global quality of life, positioning effective pain management as a critical medical priority. Currently, clinical pain treatment relies heavily on non-steroidal anti-inflammatory drugs and opioids, which, however, can lead to adverse effects and tolerance. Recent advances in nanotechnology are opening new avenues for drug delivery and therapeutic design, offering a promising area of research for developing innovative and safer pain management strategies. Unlike previous literature that primarily focuses on nanocarriers for conventional pharmaceuticals, this review systematically classifies nanomaterials according to their dual roles as precision delivery platforms and intrinsic therapeutic agents. Firstly, chronic pain analgesics are introduced through a categorization into traditional pharmacological drugs and emerging biological substances. Subsequently, an in-depth assessment of diverse nanoplatforms for drug delivery is provided. More importantly, nanomaterials possessing inherent analgesic properties are highlighted and the therapeutic mechanism is discussed. Furthermore, with an emphasis on active-targeting and stimulus-responsive release/activation strategies, the nanoplatforms for targeted pain management have been intensively introduced. Finally, this work addresses the current challenges and future perspectives of nanomedicine in pain management. By synthesizing current delivery nanoplatforms, intrinsic analgesic mechanisms of nanomaterials, the targeted pain relief strategies, and existing clinical barriers, this work aims to provide a comprehensive and informative overview of nanomaterial applications in pain management, stimulating innovative research and development within this field.

## Graphical abstract

**Figure:**
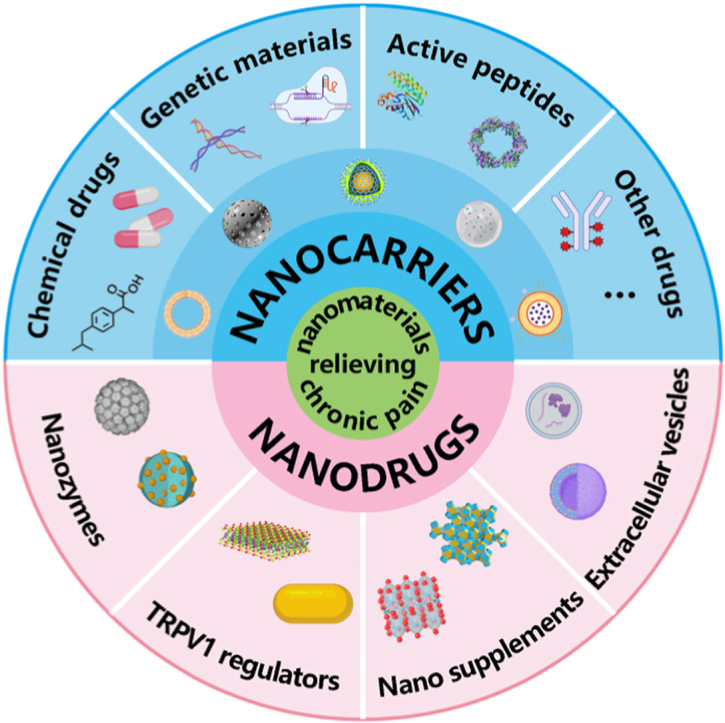

## 1 Introduction

Pain is a multifaceted unpleasant experience with sensory and emotional components, often triggered by actual or potential tissue damage [1]. In its physiological state, pain serves as a vital warning system, alerting the organism to avoid harmful stimuli, such as extreme temperatures, chemical irritants, and other noxious agents. This protective mechanism helps prevent further injury and promotes survival. However, when the underlying injury or disease persists, pain can transition from a symptom to a disease state known as chronic or pathological pain [2]. Specifically, chronic pain is defined as pain that persists or recurs for longer than three months. While mechanistically classified as nociceptive, neuropathic, or nociplastic, its diverse pathophysiology also allows for categorization into distinct phenotypes, such as inflammatory, cancer-related, and mixed pain [3]. As an incapacitating condition, chronic pain profoundly impacts quality of life, leading to significant functional impairment and diminished psychosocial well-being [4]. Global reports indicate that chronic pain affects more than 30% of the population [5]. Consequently, it represents a critical public health challenge necessitating attention and effective therapeutic approaches.

Effective pain management necessitates a comprehensive approach, acknowledging the complexity of pain treatment [[6], [7], [8]]. While non-pharmacological interventions, such as physical and psychological therapies, are essential components of a comprehensive pain management plan, the development of effective pharmacological treatments remains a critical priority [9]. By targeting the specific cause of distress, pharmacological treatments offer the most rapid, convenient, and potent means of pain control [10]. The World Health Organization analgesic ladder has historically guided pain management through a three-tiered framework [11]. It starts with non-opioid analgesics (Non-steroidal anti-inflammatory drugs (NSAIDs) and acetaminophen) for mild pain, escalates to weak opioids for moderate pain, and reserves potent opioids for severe cases. Despite its widespread use, the clinical utility of these agents is limited by their significant risk profiles. Opioids carry high potentials for addiction, respiratory depression, and overdose, while chronic use of non-opioid adjuncts like NSAIDs can result in adverse gastrointestinal and renal outcomes. To address these limitations, it is essential to develop new medications or optimize existing ones to improve their efficacy and safety [12].

**Scheme 1:**
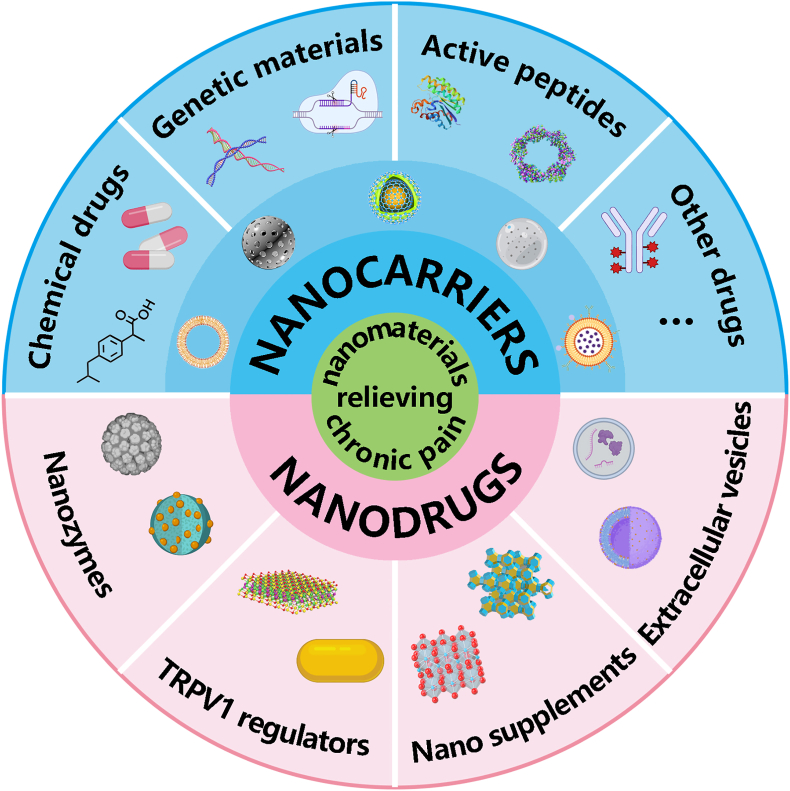
Overview of the applications of nanomaterials for chronic pain relief.

With the development of nanotechnology, nanomaterials have been explored for pain management, which may offer a promising alternative to traditional treatments [[13], [14], [15], [16]]. Attributed to their high specific surface area, biocompatibility, and unique physicochemical properties, these materials offer distinct therapeutic advantages. Specifically, they facilitate high-capacity analgesic loading to enhance local drug concentration while minimizing systemic toxicity. Furthermore, strategic surface functionalization enables nanocarriers to perform synergistic roles, such as on-demand release or multimodal combination therapy. Notably, certain nanomaterials even exhibit intrinsic analgesic properties. This review aims to provide a systematic overview of the evolving role of nanotechnology in pain modulation. While previous reviews have outlined nanomaterials for pain management, most prioritize their role as delivery platforms for conventional pharmaceuticals [[17], [18], [19]]. Furthermore, discussions regarding the intrinsic analgesic properties of nanomaterials remain fragmented, often focusing on a single material type without a comprehensive classification [20,21]. In this review (Scheme 1), we first established a baseline by discussing traditional chemical drugs and emerging biological substances. Subsequently, we examined the role of nanotechnology in pain management. This includes evaluating nanomaterials for delivery platforms and highlighting nanomaterials with inherent analgesic properties, a frequently underrepresented area of study. Furthermore, targeted therapeutic strategies of nanoplatforms against chronic pain are emphasized in terms of active targeting and stimulus-responsive release/activation. By synthesizing current delivery strategies, mechanisms of action, and remaining clinical hurdles, this review defined the state-of-the-art and identified critical pathways for the development of next-generation chronic pain interventions.

## 2 Active substances for pain relief

The landscape of pain relief is rapidly evolving past its classical origins. Analgesics can be categorized into two major groups based on their mechanism and composition. Chemical drugs act as the traditional first line of defense. They work by targeting broad inflammatory or neurological pathways throughout the body. Another class represents a shift toward precision medicine, encompassing emerging biological substances, such as genetic materials, peptides and extracellular vesicles. They employ advanced biological structures to target specific pain receptors with high accuracy.

### 2.1 Chemical drugs

Chemical drugs for pain relief, or modern pharmaceuticals, represents the primary therapeutic approach in pain management today. They have been widely used in clinical practice. These synthetic or purified compounds are designed to target and interfere with the specific biochemical pathways responsible for pain signaling in the body.

#### 2.1.1 Opioids

**Fig. 1:**
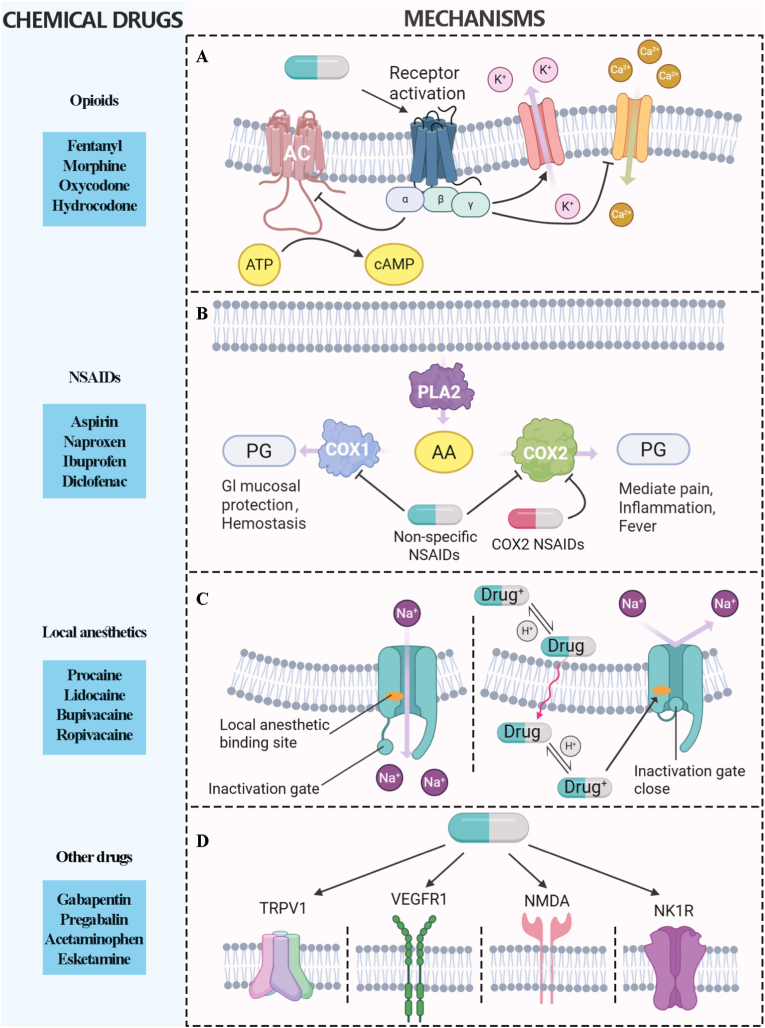
The different kind of chemical drugs and the related mechanism for pain relief.

Opioid analgesics are a powerful drug class, widely recognized as the most potent pain relievers available. It can be classified into three categories: natural opioids (derived from the opium poppy plant, such as morphine and codeine), semi-synthetic opioids (created by modifying natural opioids, such as hydrocodone and oxycodone), and synthetic opioids (man-made opioids, such as fentanyl and tramadol). The binding of opioids to opioid receptors (μ receptors, δ receptors, κ receptors, and others) triggers a cascade of events, including the reduction of intracellular cyclic adenosine monophosphate (cAMP) formation, the opening of K^+^ channels, and the suppression of Ca^2+^ channels (Fig. 1A) [22]. These events decrease intracellular Ca^2+^ availability and lead to neural hyperpolarization, and ultimately, reduce central nervous system (CNS) neurotransmitter release, leading to analgesia and mood regulation [23]. Beyond its primary function, this interaction triggers systemic side effects including nausea, emesis, and constipation [17]. Furthermore, the short half-life of many opioids necessitates frequent dosing to sustain therapeutic efficacy. This cycle often leads to tolerance. Over time, this necessity for higher dosing can result in physical dependence and withdrawal symptoms [24]. Accordingly, elevating the targeting specificity and prolonging the half-life of opioid agents offers a viable means for reducing their adverse reactions.

#### 2.1.2 NSAIDs

NSAIDs are among the most widely prescribed therapeutic agents globally, serving as the primary pharmacological strategy for managing mild-to-moderate pain, inflammation, and fever. Their popularity stems from their established efficacy and a more favorable side-effect profile compared to steroidal alternatives. The mechanism of action for NSAIDs is well-defined. They selectively target cyclooxygenase (COX) enzymes to suppress the production of prostaglandins, which drive the inflammatory and nociceptive response [25]. This targeted inhibition effectively reduces symptoms and provides necessary pain relief (Fig. 1B). However, the therapeutic utility of these drugs is fundamentally compromised by significant, dose-dependent adverse effects associated with long-term use, particularly gastrointestinal damage, cardiovascular risks, and renal toxicity. Furthermore, the inherent poor water solubility of many NSAID compounds necessitates high dosing to achieve effective therapeutic concentrations, which further exacerbates these side effects. Therefore, advancing the efficacy and safety of NSAIDs by developing novel formulations that improve their solubility and bioavailability is an essential objective for the future of pain management.

#### 2.1.3 Local anesthetics

Local anesthetics (LAs), encompassing widely utilized drugs such as lidocaine, bupivacaine, and ropivacaine, are fundamental agents in pain management and regional anesthesia. Their potent analgesic effect is achieved through a localized action. LAs function by reversibly inhibiting voltage-gated sodium channels (Na_V_) on neuronal membranes. By preventing the influx of sodium ions, LAs stabilize the neuronal membrane, which effectively blocks the initiation and propagation of action potentials [26]. This mechanism interrupts the transmission of pain signals from the peripheral site to the CNS, providing immediate and profound analgesia (Fig. 1C). Despite their high efficacy, the clinical utility of LAs is mainly constrained by two limitations: their short duration of action requiring repeated administration, and the significant risk of systemic toxicity. Excessive systemic absorption can lead to high plasma concentrations, resulting in dose-dependent adverse effects, particularly severe cardiovascular toxicity (such as arrhythmia and cardiac arrest) and neurological toxicity (including seizures and CNS depression). Consequently, developing delivery systems to prolong their local action while rigorously minimizing systemic exposure is a major challenge in modern anesthesiology [27].

#### 2.1.4 Other chemical drugs

Beyond the above drugs, several other classes of chemical drugs are essential in pain management, particularly for chronic and neuropathic pain (Fig. 1D). These medications include anticonvulsants, such as gabapentin and pregabalin. While originally developed for seizures, they are now highly effective for nerve pain. They work by quieting overactive nerve signals in conditions like diabetic neuropathy or postherpetic neuralgia [28]. Similarly, certain antidepressants, especially tricyclic antidepressants and serotonin-norepinephrine reuptake inhibitors, are utilized for their analgesic properties. They work by increasing the levels of certain neurotransmitters in the spinal cord that help inhibit pain signals traveling to the brain [29].

Additionally, the common over-the-counter pain reliever acetaminophen is a distinct non-opioid analgesic that is not classified as an NSAID. It primarily acts centrally in the brain and spinal cord to block the formation of prostaglandins, which are key chemical mediators of pain, and is widely used for mild to moderate pain and fever [30]. Furthermore, muscle relaxants can be used to treat pain associated with muscle spasms, and corticosteroids may be injected near nerves or into joints to reduce inflammation and pain [31]. These varied agents demonstrate a multi-faceted pharmacological approach required to effectively target different mechanisms of pain across various conditions.

### 2.2 Biological substances

**Fig. 2:**
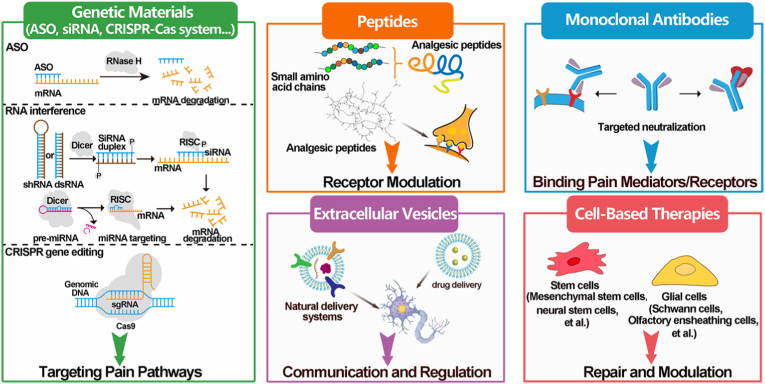
The emerging biological substances for pain management.

In contrast to traditional chemical drugs, the cutting edge of pain management is increasingly defined by emerging biological substances that move beyond simple systemic chemical intervention. This next generation of treatments represents a paradigm shift from broad pharmacological relief to targeted, regenerative, or even curative strategies (Fig. 2). These modalities include advanced biomolecules designed to specifically modulate pain pathways, cell-derived vesicles capable of bypassing physiological barriers to deliver therapeutic payloads, and cell-based therapies aimed at repairing damaged tissue or modulating the pain-sensing environment. While some of them have already transitioned into clinical utility, others remain under preclinical or fundamental investigation. Ultimately, these substances offer the potential for enhanced efficacy and reduced systemic toxicity, providing a novel framework for treating intractable pain conditions refractory to current standards of care.

#### 2.2.1 Genetic materials

Gene therapy is a medical treatment that involves introducing genetic material into cells to prevent or treat diseases [[32], [33], [34]]. The genetic material used in gene therapy can comprise DNA, RNA, or gene editing tools, which enable gene replacement, gene silencing, and gene editing, thereby modulating gene expression to achieve therapeutic effects. In the context of chronic pain management, gene therapy offers several potential advantages [35]. By introducing genes that code for pain-relieving proteins or peptides, or by targeting genes that contribute to pain, gene therapy can provide long-term pain relief, targeted therapy, sustained release, and personalized medicine, while minimizing the risk of systemic side effects and addiction [36]. For instance, antisense oligonucleotides work by binding to messenger RNA (mRNA) to prevent the production of the targeted protein or peptide, effectively silencing the gene. It had been studied for treating pain, such as neuropathic pain, where certain ion channels (Na_V_, voltage-gated calcium channel (Ca_V_), transient receptor potential, etc.) or receptors (purinergic receptor 2X, N-methyl-d-aspartate (NMDA) receptor, etc.) became upregulated or hyperfunctional after nerve damage [37,38]. Similarly, small interfering RNA (siRNA), selective downregulation of target mRNA to abolish the pain-related behaviors/pathophysiological pain response, have also been researched [39]. For instance, the siRNA for transient receptor potential A1 (TRPA1) had been used to ameliorate chemotherapy-induced peripheral neuropathy [40]. Furthermore, as a prominent gene editing tool, the CRISPR-Cas9 system is recognized for its potential in gene therapy, have shown therapeutic efficacy in multiple animal models of human diseases, including pain [41]. Although promising, several candidates remain in clinical trials for common chronic pain conditions and the translation of them is currently restricted [42]. Furthermore, delivery remains a significant hurdle. These molecules are not only highly susceptible to degradation, but their substantial size and negative charge also impede cellular uptake. In addition, the immune system may recognize genetic materials, triggering an immune response that can reduce their efficacy and increase side effects. To overcome these challenges, a carrier is essential to protect genetic materials from degradation, target their delivery to specific cells or tissues, facilitate cellular uptake, reduce immunogenicity, and stabilize them for storage and transport, ultimately enabling the safe and effective delivery of genetic materials into cells [43].

#### 2.2.2 Peptides

Peptides offer a rapid and direct therapeutic approach to pain relief compared to the sustained, but slower, effects of gene therapy [44,45]. These molecules are short chains of amino acids, falling in a unique size range, larger than traditional small-molecule drugs but smaller than monoclonal antibodies, that grants them high specificity. They function by selectively and potently targeting critical components of the pain signaling cascade, such as specific ion channels and receptors on the surface of pain-sensing neurons (nociceptors) [46]. With these unique therapeutic advantages, peptides have undergone extensive investigation, with several successfully translated into clinical practice. A powerful example is ziconotide (Prialt), a synthetic peptide originally derived from cone snail venom. It functions by blocking Ca_v_2.2 in dorsal horn sensory neurons, thereby inhibiting the release of pro-nociceptive neurotransmitters within the spinal cord [47]. It has been used for severe chronic pain. However, its administration is limited to the intrathecal route, as ziconotide cannot cross the blood-brain barrier and triggers severe neurotoxicity if injected systemically. In addition, there are various other therapeutic peptides for chronic pain under fundamental research. For instance, Gao's group found that the follistatin (FST)-Insulin-like growth factor-1 receptor signaling axis was a major cause of nerve cell overactivity in chronic pain [48]. They also developed peptides derived from the N-terminal of FST. Notably, one candidate successfully attenuated Na_V_1.7-mediated hyperexcitability in nociceptive neurons, effectively reducing pain behaviors in a murine model of neuropathic pain. Despite their potential, native peptides require advanced engineering to overcome challenges such as poor oral bioavailability, low membrane permeability, and rapid *in vivo* degradation and clearance. To overcome these challenges, efforts are focused on enhancing peptide stability, extending their circulation time, and facilitating sustained, localized release, all of which are critical for optimizing their analgesic efficacy.

#### 2.2.3 Monoclonal antibodies

Monoclonal antibodies (mAbs) are a class of advanced biomolecules engineered as large, Y-shaped protein molecules designed to bind to a single, specific target protein with exceptionally high affinity. In pain management, they have been utilized as targeted therapies to block key inflammatory or neurotrophic factors that drive chronic pain, offering a highly precise mechanism of action. Due to their large molecular size, they generally do not cross the blood-brain barrier (BBB), thereby minimizing the CNS side effects common in traditional small-molecule drugs. A variety of mAbs are currently in development, given their capacity to target a broad spectrum of molecules implicated in pain signaling transmission and chronification. Target molecules include pleiotropic cytokines such as tumor necrosis factor (TNF) and nerve growth factor (NGF), receptor tyrosine kinases represented by epidermal growth factor receptor, interleukins (e.g., IL-6, IL-20) and their corresponding receptors, among many others [49]. These interventions aim to manage chronic pain across several etiologies, including osteoarthritis (OA), rheumatoid arthritis (RA), and neuropathic or low back pain. For instance, mAbs targeting NGF, such as tanezumab and fasinumab, function by physically neutralizing circulating NGF. This prevents the signaling protein from activating receptors on peripheral nociceptors, demonstrating considerable efficacy in managing chronic OA and low back pain [50]. Beyond these emerging targets, several mAbs have already reached clinical application. Most notably, four mAbs targeting calcitonin gene-related peptide (CGRP) or its receptor are now approved for migraine treatment. CGRP is a neuropeptide produced in both the CNS and peripheral nervous system that modulates nociceptive transmission by activating mitogen-activated protein kinase and receptor activity-modifying protein 1 [51]. However, the aforementioned inability of mAbs to penetrate BBB is also a limitation. For a better understanding of the role mAbs play in central sensitization and enhance their therapeutic potential within the CNS, utilizing specialized delivery platforms could help. Furthermore, these platforms may enable targeted local delivery, prolonged drug retention, and sustained release, thereby optimizing the efficacy of mAbs in chronic pain management.

#### 2.2.4 Extracellular vesicles

Extracellular vesicles (EVs) are lipid-bound vesicles released by cells into the extracellular space, comprising lipids, nucleic acids, and proteins [52]. They mediate cell signaling and signal transduction, modulating pivotal biological events including tissue repair and immune regulation [53]. There are mainly three subtypes of EVs, including exosomes, microvesicles, and apoptotic bodies [54]. Exosomes, typically 30-150 nm in diameter, are formed through the inward budding of multivesicular bodies and subsequent fusion with the plasma membrane. In contrast, microvesicles, which range from 100 to 1000 nm in diameter, are formed directly from the plasma membrane through budding. Apoptotic bodies, formed during late-stage apoptosis, are larger membrane-bound vesicles (diameter >1000 nm) that carry cellular remnants and molecular signals [55]. As it has become clear that most methods used to isolate exosomes co-isolate heterogeneous populations of EVs of diverse biogenic origin, EVs with diameter less than 200 nm have been defined as small extracellular vesicles (sEVs), which are currently the most researched population [56]. Given their biogenic origin, biodegradability, and ability to traverse biological barriers, such as the blood-brain and blood-spinal cord barriers, sEVs have emerged as a promising platform for biological applications [57,58]. The sEVs from diverse sources have shown therapeutic potential in alleviating pain and other diseases [[59], [60], [61], [62], [63], [64], [65], [66]]. Notably, sEVs from various mesenchymal stem cell sources, including bone marrow, induced pluripotent, umbilical cord, placenta, and others, have demonstrated remarkable pain-relieving efficacy [[67], [68], [69], [70], [71]]. The mechanism is primarily attributed to the proteins and miRNAs within EVs, which suppress neuron and glial activation, thereby inducing anti-inflammatory and analgesic effects. For example, Rong's group explored the therapeutic potential of bone marrow mesenchymal stem cell-derived exosomes in OA. Their findings showed that the exosomes effectively promoted cartilage repair and extracellular matrix synthesis, and significantly alleviated knee pain in OA rats [72]. Despite these promising results, no EV-based therapies for chronic pain have yet received regulatory approval. Consequently, research is increasingly focusing on engineered EVs to improve yield, targeting precision, and overall therapeutic potency [73].

#### 2.2.5 Cell secretome

Beyond EVs, cells also secrete a diverse array of soluble bioactive substances into the extracellular microenvironment, and all these secreted components collectively constitute the cell secretome. The secretome encompasses not only soluble proteins, such as cytokines, chemokines, and growth factors, but also bioactive lipids, microRNAs (miRNAs), and EVs. Notably, the secretome from stem cells has shown facilitate physiological and therapeutic effects *via* mechanisms such as intercellular communication, immunomodulation, neuroprotection, and tissue regeneration [74]. By serving as a cell-free proxy for stem cell therapy, the secretome offers a safer, more controllable alternative to live cell transplantation, reducing risks associated with unwanted differentiation or malignant transformation. These properties have been extensively explored across a variety of pathologies, with particularly promising results in the management of chronic pain [75]. Among diverse stem cell sources, the mesenchymal stem cell (MSC) secretome, specifically from umbilical cord, adipose, bone marrow, and placental tissues, possesses the strongest evidence for treating chronic pain [76]. It attenuates neuropathic, inflammatory, diabetic, and osteoarthritic pain by suppressing pro-inflammatory cascades, inhibiting glial overactivation, and modulating pain signaling pathways [[77], [78], [79]]. While veterinary-grade lyophilized MSC products are currently used for equine and canine OAs, human applications remain unapproved. Widespread clinical translation is currently hindered by batch-to-batch heterogeneity, a lack of standardized quality control, and evolving regulatory frameworks.

#### 2.2.6 Cell-based therapies

While the scientific community increasingly favors cell-free therapies for their safety and scalability, whole-cell therapy offers unique biological advantages that isolated components cannot replicate. By acting as dynamic pharmacies, living cells sense specific microenvironmental cues and adaptively release therapeutic factors in real-time. This represents a paradigm shift in chronic pain management, moving beyond conventional pharmacology, which often merely masks symptoms, toward a responsive and biological intervention. This category involves the transplantation or injection of living cells, either autologous (from the patient) or allogeneic (from a donor), into the body with the goal of modulating the biological environment, repairing damaged tissue, or directly interfering with pain signaling pathways [80]. One kind of the cells are MSCs, which can differentiate into various cell types, secrete growth factors or anti-inflammatory factors that promote the repair of damaged cartilage, bone, or neural structures implicated in musculoskeletal or neuropathic pain [81]. A key advantage of MSC therapy is its low immunogenicity, which allows the body to accept the injection without a major immune rejection [82,83]. Additionally, neural stem cells (NSCs) are self-renewing multipotent cells capable of differentiating into neurons, astrocytes, and oligodendrocytes. When transplanted, they can alleviate neuropathic pain by restoring neural connectivity and providing antioxidant, anti-inflammatory, and anti-matrix degradation benefits [84]. Furthermore, the glial cells, such as Schwann cell and Olfactory ensheathing cells have also been studied with pain relief function, by down-regulating the expression of pain-related molecules [85,86]. This area is rapidly evolving, holding significant promises for patients suffering from conditions like severe OA, chronic low back pain, and certain forms of neuropathic pain where current treatments fall short.

## 3 The applications of nanomaterials for pain relief

Advances in nanotechnology are transforming the landscape of pain management. By leveraging the unique properties of nanomaterials, researchers are developing innovative strategies that offer significant advantages over traditional analgesic therapies. Specifically, nanoparticles (NPs) have emerged as powerful tools with a remarkable dual functionality in alleviating pain. On one hand, they act as highly efficient delivery systems for a wide array of pain-relieving pharmaceuticals, enhancing their bioavailability and targeting specific sites of action. On the other hand, certain NPs possess intrinsic analgesic properties, offering a therapeutic effect even without a traditional drug payload. The following section will thoroughly delve into both critical roles, examining how NPs function as advanced drug carriers and exploring the mechanisms behind their inherent pain-relieving capabilities.

### 3.1 NPs as drug delivery platform

Owing to their biocompatibility, porous structures, and high surface area, NPs serve as versatile drug delivery platforms. The unique physicochemical properties allow NPs to overcome the pharmacokinetic limitations of traditional analgesics by enhancing both precision and stability. By encapsulating hydrophobic or easily degradable compounds, NPs improve drug solubility and bioavailability while protecting potent treatments from premature degradation. In addition, NPs facilitate a sustained-release profile to maintain steady drug concentrations and possess the specialized ability to bypass formidable physiological hurdles, such as the BBB, to treat centralized chronic pain. To date, a variety of NPs have been developed to deliver chemical drugs and biological substances for pain management (Table 1). By utilizing NPs, analgesics can achieve improved solubility, an elongated half-life, and sustained release, thereby enhancing their overall therapeutic efficacy.

**Table 1:** The various drug delivery nanoplatforms for chronic pain therapy.

Types	Nanomaterials	Size	Routes of administration	Pain model	Encapsulated drugs	Loading efficiency	Analgesic time	Reference
Lipid-related NPs	TRPA1 siRNA-entrapped liposomes	198.9 ± 1.62 nm	Intrathecal injection; Intravenous injection	Chemotherapy-induced peripheral neuropathy	TRPA1 siRNA	EE: 92.6 ± 3.5%	5 days	[40]
	PLA NPs SLNs NLCs	113.7 ± 4.1 nm	Topical administration	-	Tetracaine	EE: >85%	3.5 h-8 h	[87]
	sConA-Lip/TD	Around 500 nm	Topical instillation	Corneal tactile sensitivity model	Tetrodotoxin Dexmedetomidine	TTX: >43% DMED: >62%	10 h	[88]
	TRPsiRNA-SLNs	103.0 nm ± 0.1 nm	Topical administeration; Intraplantar administeration	Thermal hyperalgesia model; Capsaicin-induced pain model and nocifensive pain model	TRPsiRNA	EE: 98.51 ± 1.63%	Topical administration: 24h; Intraplantar administration: 120h	[89]
Polymeric NPs	K-CS NPs	232.7 ± 4.5 nm	Intra-articular injection	-	Celecoxib Kartogenin	CE: 70.5 ± 2.2%	-	[90]
	Que-Mg@SA NPs	Around 187 nm	Intra-articular injection	-	Que-Mg	LE: 10.17 ± 0.51%	-	[91]
	NSTX-AlgPLGA-MPs	7.6 ± 4.0 μm	Intra-articular injection	Destabilization of the medial meniscus mouse model; Partial meniscectomy mouse model; Anterior cruciate ligament transection rat model	Neosaxitoxin	LC: 2.4 ± 0.1%	2 weeks	[92]
	EM/siKEAP1@Fe-MOF@HA	Around 207.5 nm	Intravenous injection	A collagen-induced RA model	Emodin; siKEAP1	Emodin: 23.85%; siKEAP1: 9.85%	15 days	[93]
	HCPC/DEX NPs	89.73 ± 18.89 nm	Intravenous injection	Adjuvant-induced Arthritis	Dexamethasone	LC: 4.79%	15 days	[94]
	PEI	-	Intrathecal injection	Chronic constriction injury of the sciatic nerve	TRPV1 siRNA	-	5 days	[95]
	PLGA NPs	Around 227.8 nm	Intrathecal injection	Spinal Nerve Ligation	CX3CR1 siRNA	-	14 days	[96]
	PLGA NPs	Around 247.5 nm	Intrathecal injection	Spinal Nerve Ligation	IKBKB siRNA	-	8-10 days	[97]
	PLGA NPs	224 ± 16.5 nm	Intrathecal injection	Spinal Nerve Ligation	pFoxp3	-	7 days	[98]
Metal-Organic framework NPs	Fe_3_O_4_@ZIF-8-RVC	192.3 ± 71.2 nm	Intravenous injection	Chronic constriction injury rat model	Ropivacaine	LC: 20.3 ± 0.26%	24 h	[99]
	Bai@FA-UIO-66-NH_2_	283.4 ± 10.03 nm	Intra-articular injection	ACLT-induced OA model in SD rats	Baicalin	-	8 weeks	[100]
	Ce-UiO-66-Bupivacaine	Around 156.9 nm	Local injection	Chronic constriction injury model of the sciatic nerve in mice	Bupivacaine	LC: 85%	36 h	[101]
Mesoporous silica	MCM-41 nanoparticles	Around 160 nm	-	-	Ibuprofen	LD: 25.50 ± 0.57%	-	[102]
	MSN-COOH	Around 238.6 nm	Intragastric administration	λ-carrageenan-induced paw edema model	Nimesulide; Indomethacin	NMS: LC: 25.3 ± 0.1% IMC: LC: 34.0 ± 0.7%	>4 h	[103]
	miR-26a-5p@A-MSN-MG1	Around 150 nm	Intrathecal injection	SNI-induced neuropathic pain models; CFA-induced inflammatory pain; paclitaxel-induced CIPN models	miR-26a-5p	LC: 78.54%	SNI: 42 days; CFA: 14 days; CIPN: 9 days	[104]
Injectable nano-composite hydrogels	DAGQD@Cu@KGN-SO_3_^-^/DA-HA	5-10 nm	Intra-articular injection	Collagen-induced arthritis rat model; Ovalbu-min induced arthritis rabbit (male) model	Kartogenin	LC: 1.1%	CIA: 4 weeks OIA: 8 weeks	[105]
	CLDAFR	25–30 nm	In situ injection	Chronic constriction injury of brachial plexus	Ropivacaine Celecoxib	Ropivacaine: 80% Celecoxib: 90%	14 days	[106]
	PNSH	62.41 ± 0.84 nm	In situ injection	Rat RA model	Methotrexate	LC: 1.3%	28 days	[107]
	OSPPB	Around 424 nm	In situ injection	A unilateral crossbite mouse model	Bevacizumab	-	3 weeks	[108]
sEVs and Viral vectors	Hypo-VSC EVs	129.1 ± 60.5 nm	Intrathecal injection	Ischaemia-reperfusion-induced pain	miR-126-3p	-	Up to 72 h	[73]
	HSV-ENK	-	In situ injection	Formalin inflammatory pain model	Enkephalin	-	10 days	[109]
	HSVLatEnk	-	In situ injection	Chronic constrictive injury of the infraorbital nerve	Proenkephalin A	-	5 weeks	[110]
	DCES	247 ± 120.3 nm	Intramuscular injection	Streptozotocin-induced rat model of diabetic peripheral neuropathy	BMSCs; PpyNPs	BMSCs: 28%	8 weeks	[111]
	KLS-2031	-	Transforaminal epidural injection	Spared nerve injury	GAD65; GDNF; IL-10	-	12 weeks	[112]
	AAV9	-	Intrathecal injection	Carrageenan-induced inflammatory pain; Paclitaxel-induced neuropathic pain; BzATP-induced pain	ZFP-KRAB; KRAB-dCas9	-	10 months; 3.5 months	[113]

#### 3.1.1 Lipid-related NPs

**Fig. 3:**
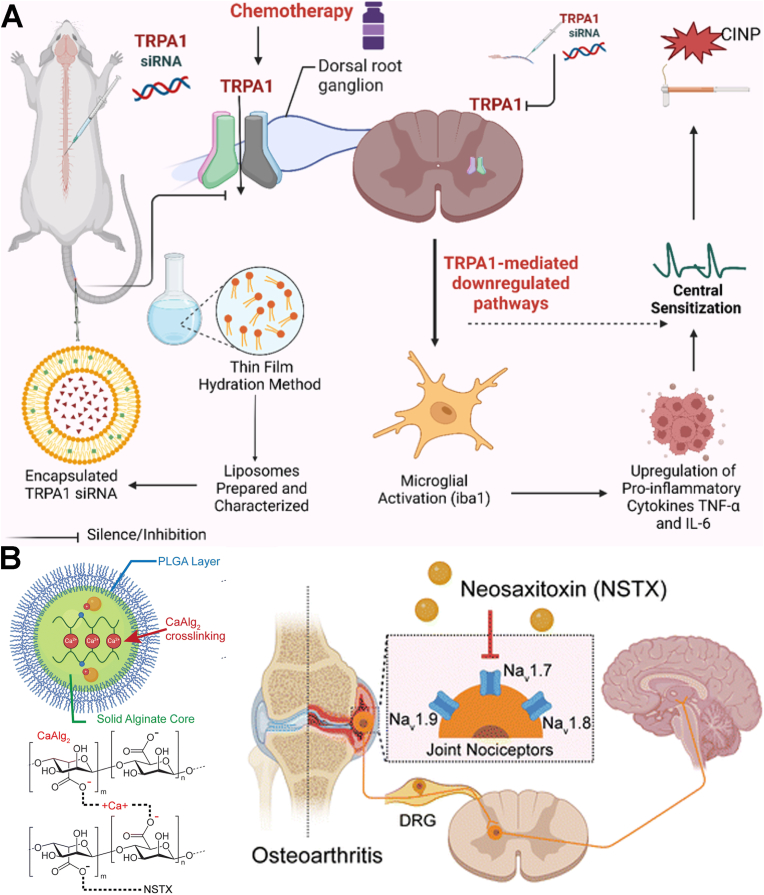
(A) Schematic illustration of TRPA1 siRNA-encapsulated cationic lipids for the systemic treatment of CINP. Reprinted with permission from Ref. [40]. Copyright America Chemical Society 2025. (B) Schematic illustration of the construction of neosaxitoxin delivered alginate-PLGA microparticles and the analgesic mechanism for OA. Reprinted with permission from Ref. [92]. Copyright Royal Society of Chemistry 2025.

Lipid-related NPs are primarily classified into four categories based on their lipid matrix composition and internal structure: liposomes, solid lipid NPs (SLNs), nanostructured lipid carriers (NLCs) and lipid NPs (LNPs). The differences between these four nanocarriers primarily lie in their internal architecture and the physical state of their lipid components. Liposomes are characterized by one or more phospholipid bilayers surrounding an internal aqueous core, making them ideal for carrying both hydrophilic and lipophilic compounds. In contrast, SLNs utilize a solid crystalline lipid matrix instead of an aqueous core, ensuring better stability and more precise release for hydrophobic drugs. To address the limited drug-loading capacity and potential leakage issues of SLNs, NLCs utilize a mixture of solid and liquid lipids to create an imperfect matrix that offers more space for therapeutic molecules. Finally, LNPs are distinct from the others as they typically utilize ionizable lipids to form complex, non-vesicular structures specifically designed for the protected delivery and cellular uptake of nucleic acids like mRNA. These lipid-related NPs, have emerged as highly effective delivery platforms, characterized by their ability to stabilize payloads and extend drug release profiles [114,115]. Their natural biodegradability ensures that these systems can be safely metabolized and eliminated, significantly reducing the risk of systemic toxicity. Lipid-related NPs have been utilized as carriers for pain-relief drugs, including opioids, NSAIDs, and LAs [87]. Several related formulations have been authorized by the food and drug administration (FDA) for pain management [116,117]. For instance, encapsulating bupivacaine hydrochloride within liposomes allows the nanoformulation to release the drug consistently over an extended period (72-96 h). This product, Exparel, has been used for postoperative pain management *via* a single local infiltration at the surgical site or through specific nerve blocks. In addition, the versatility of modification on lipid-related NPs facilitates targeted delivery. For example, Kohane's group synthesized succinyl-Concanavalin A-modified liposomes loaded with tetrodotoxin and dexmedetomidine [88]. These liposomes bound to corneal glycan moieties, enabling the targeted and sustained release of both drugs. Consequently, topical administration provided prolonged corneal anesthesia without impairing the wound-healing process. Apart from chemical drugs, lipid-related NPs have also been studied for delivering siRNA for pain relief [89]. For instance, Tiwari's group utilized cationic lipids to deliver TRPA1-targeting siRNA to treat chemotherapy-induced neuropathic pain (CINP) (Fig. 3A). As a polymodal and nonselective cation channel, TRPA1 is activated by diverse stimuli and cellular stress products. Its overexpression in the dorsal root ganglia (DRG) and spinal cord during CINP pathogenesis exacerbates neuropathic pain. Following intrathecal administration, this nanoformulation downregulated TRPA1 mRNA and protein expression in the DRG and spinal cord, significantly reducing mechanical and cold hypersensitivity [40]. As clinically established drug delivery platforms, lipid-based NPs offer numerous advantages, including excellent biocompatibility, low immunogenicity, scalable preparation, and the flexibility to load both hydrophobic and hydrophilic agents for sustained release. Despite the clinical success of some lipid-based nanocarriers, their variety remains limited. Significant challenges persist regarding targeting precision, BBB penetration, and *in vivo* stability. Furthermore, issues such as low loading capacity, premature burst release, and the lack of sophisticated targeting strategies for chronic pain-related receptors, highlight that this field is still in its developmental stages.

#### 3.1.2 Polymeric NPs

Polymeric NPs are synthesized from natural or synthetic monomers or polymers, allowing for the engineering of intelligent systems with tailored structures [118]. These polymers are broadly categorized by their origin: natural (e.g., chitosan, gelatin, hyaluronic acid, and alginate) or synthetic (e.g., poly(lactic-co-glycolic acid) (PLGA), poly(amidoamine) (PAMAM), poly(lactic acid) (PLA), poly(L-lysine) (PLL), poly(ε-caprolactone) (PCL), and polyanhydrides.). Due to their inherent biocompatibility and biodegradability, natural polymers are often the preferred scaffold for drug delivery. Chitosan, a chitin-derived polysaccharide, is characterized by its biocompatibility, biodegradability, and potent antimicrobial activity. Beyond its capacity to accelerate tissue regeneration *via* fibroblast proliferation, collagen deposition, and angiogenesis, it exerts immunomodulatory effects that mitigate excessive inflammation. When formulated as NPs, chitosan exhibits superior solubility and mechanical strength, making it a promising vehicle for drug delivery in wound healing and OA [119,120]. For instance, Nasrabadi's group prepared celecoxib and kartogenin loaded chitosan NPs [90]. Injecting these two NPs into the knee cavity improved drug bioavailability, rapidly suppressed inflammation and pain, and promoted cartilage regeneration. Alginate is an FDA-approved polymer characterized by a backbone rich in free carboxyl and hydroxyl groups. The development of derivatives from these functional groups enhances solubility, mitigates systemic toxicity, and increases drug affinity. Consequently, alginate NPs are widely utilized as biodegradable and biocompatible carriers for anti-inflammatory and anti-tumor agents. They have also been used for carriers for OA treatment by enhancing the therapeutic profile and safety of drugs, such as fucoxanthin and quercetin [91,121]. In addition, the microparticles consisted with alginate core and PLGA layer were prepared by Bajpayee's group to deliver neosaxitoxin (NSTX) for extended OA pain relief (Fig. 3B) [92]. NSTX is a potent site-1 sodium channel blocker with a high affinity for Na_V_1.7. While clinical studies show that subcutaneous NSTX safely blocks sensory pain in humans, its short half-life limits its utility for treating OA. To address this, the group developed a delivery system featuring a negatively charged alginate core, which enhances NSTX encapsulation and minimizes burst release *via* electrostatic interactions. In murine models, intra-articular injection of these particles extended pain relief to one week. Joint retention was further improved by functionalizing the particle surface with cationic avidin. This charge reversal facilitates electrostatic binding to the anionic synovial matrix, preventing rapid clearance. Besides, hyaluronic acid is a natural glycosaminoglycan that binds specifically to CD44 receptors, a property that facilitates its use in targeted drug delivery systems for anticancer therapies. Hyaluronic acid-based NPs offer several advantages, including efficient targeting, tunable particle size, and stimuli-responsive drug release (triggered by pH or hyaluronidase). Furthermore, hyaluronic acid is highly biocompatible, biodegradable, and stable, with a versatile capacity for loading various therapeutic agents. Given that the immune system mediates the bidirectional crosstalk between pain and inflammation, hyaluronic acid NPs can be engineered to target macrophages. By loading these systems with antinociceptive and anti-inflammatory drugs, they can effectively localize treatment to relieve pain and enhance overall therapeutic efficacy toward RA [93,94].

Compared to naturally sourced polymers, synthetic polymers offer superior scalability, cost-effectiveness, and highly tunable physicochemical properties. Among these, PLGA and PAMAM are widely utilized for NPs synthesis due to simple preparation, biodegradability, and high drug-loading capacity. PLGA NPs have been used for the delivery of opioids [122,123]. These nanoplatforms all showed prolonged drug release and extended analgesia when compared with the free drugs. They have also been extensively explored as a delivery platform for gene therapy, successfully delivering siRNAs targeting TRPV1, the inhibitor of NF-κB kinase subunit beta, and CX3CR1, as well as Foxp3-expressing plasmid. All of these therapeutics have exhibited prominent analgesic effects [[95], [96], [97], [98]]. Unlike PLGA, PAMAM NPs are dendrimers, highly branched, three-dimensional spherical macromolecules. Drug loading within PAMAM NPs primarily relies on a combination of internal encapsulation and surface conjugation. Beyond improving the solubility of chemical drugs and the delivery of antibody, PAMAM NPs are particularly effective for gene delivery due to their repetitive branching and variable charge [124,125]. However, their clinical utility is often hindered by cytotoxicity and low transfection efficiency. These limitations are typically addressed through surface modification or by altering the dendrimer generation.

Generally, polymeric NPs offer distinct advantages for chronic pain therapy, specifically regarding their structural stability, high drug loading, and tunable physicochemical properties. Compared to lipid-based NPs, they are more easily engineered for stimuli-responsive release and surface modification, making them adaptable to various administration routes for sustained analgesic delivery. However, their clinical translation remains hampered by certain inherent limitations. The degradation by-products of specific polymers can trigger neuroinflammation, and their ability to penetrate BBB is often inferior to lipid-based systems. Furthermore, complex preparation protocols, poor batch-to-batch consistency, and a lack of long-term biosafety data continue to hinder their widespread clinical application.

#### 3.1.3 Metal-organic framework NPs

Metal-Organic Frameworks (MOFs) are crystalline materials defined by extraordinary porosity and vast internal surface areas. These networks extend into 1D, 2D, or 3D structures where inorganic metal nodes are bridged by organic ligands. Currently, MOF NPs are gaining significant traction in the biomedical field as versatile carriers for chemotherapeutic drugs, genes, proteins, and other biomacromolecules [126,127]. They have also been studied as delivery platform for pain relief. For instance, Li's group prepared ropivacaine (Ropi) and Fe_3_O_4_ encapsulated ZIF-8 nanocomposite, which leveraged the magnetic property of Fe_3_O_4_ and the acid-responsive decomposition of ZIF-8 to achieve precise targeting and controlled release of Ropi [99]. The nanocomposite exhibited high drug loading, sustained release in acidic environments, and excellent biocompatibility, resulting in significantly extended analgesic effects in a chronic constriction injury (CCI) rat model while minimizing systemic exposure. Furthermore, unlike traditional nanocarriers, MOFs offer unprecedented structural customization, allowing researchers to precisely tune physicochemical properties for targeted therapies. For example, Yin's group developed a baicalin delivery system for OA treatment using folic acid-modified UiO-66-NH_2_ [100]. By leveraging its outstanding biocompatibility, high porosity, and surface reactivity, this system efficiently delivered baicalin into macrophages to scavenge reactive oxygen species (ROS) and promoted M1-to-M2 macrophage polarization to alleviate chronic OA pain.

**Fig. 4:**
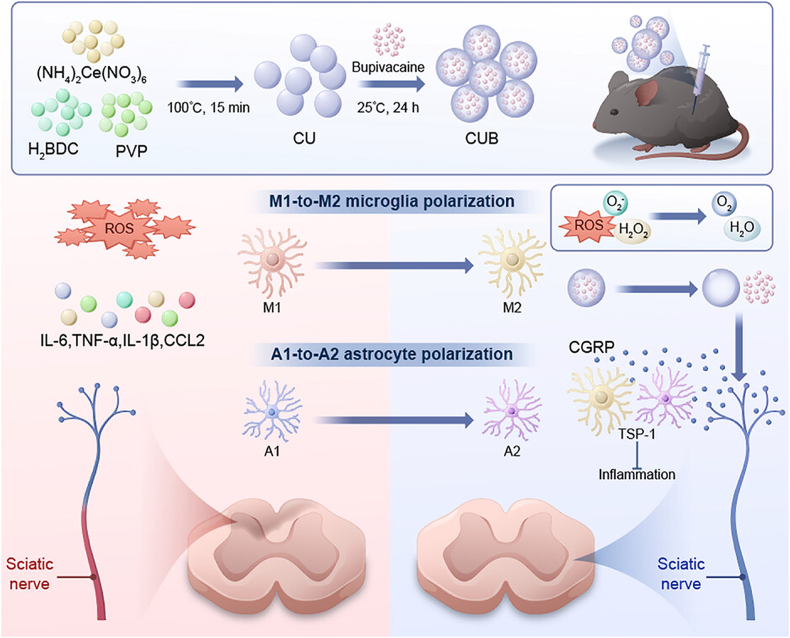
Schematic illustration of the preparation process of bupivacaine loaded Ce-UiO-66 (CUB) and the proposed mechanisms of CUB for treating neuropathic pain. Reprinted with permission from Ref. [101]. Copyright Wiley 2025.

Beyond serving as delivery vehicles, MOFs can also integrate secondary therapeutic functions to synergistically enhance analgesia. For instance, Jiang et al. developed a dual-action system (CUB) using cerium-based MOFs to deliver bupivacaine for the treatment of neuropathic pain. [101]. This scaffold combined ROS-scavenging properties with pH-responsive drug release. By promoting M2 microglial and A2 astrocyte polarization, CUB effectively suppressed neuroinflammation and facilitated myelin regeneration, leading to sustained analgesia (Fig. 4). Mechanistically, CUB drove nerve repair by upregulating thrombospondin-1 and activating CGRP signaling. Ultimately, CUB's integrated approach to pain relief and motor recovery significantly outperformed bupivacaine or MOF monotherapies. It offered a novel strategy for MOF-based neuropathic pain therapy. Despite these impressive achievements in the field, critical aspects of MOFs, such as their drug loading/release kinetics, *in vivo* toxicity, degradation mechanisms, and pharmacokinetics still need detailed investigation.

#### 3.1.4 Mesoporous silica

Mesoporous silica NPs (MSNs) are inorganic scaffolds defined by an ordered SiO_2_ framework [128]. They are characterized by diverse morphologies, uniform pore sizes, large specific surface areas, and adaptable surface chemistry. Notably, amorphous silica (the main component of MSNs) has been recognized as safe by both the FDA and the European Food Safety Authority, underscoring the good biocompatibility of MSNs. These attributes enable MSNs to serve as versatile vehicles for drug delivery, bioimaging, and biosensing. Studies have shown that MSNs can deliver poorly water-soluble drugs, such as NSAIDs. For instance, MSNs were reported to encapsulate ibuprofen with up to 80% loading efficiency, allowing for sustained drug release for up to 48 h [102]. In addition to carrying a single analgesic, MSNs have also been used to carry multiple drugs. For example, carboxyl groups functionalized MSNs have been shown to significantly enhance the dissolution of nimesulide and indomethacin, leading to improved anti-inflammatory and analgesic effects [103].

**Fig. 5:**
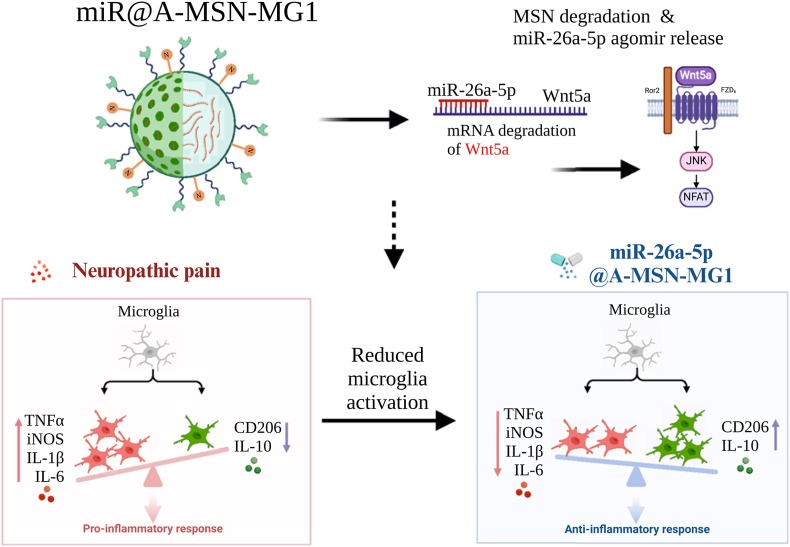
Schematic representation of miR-26a-5p loaded, MG1-functionalized MSNs. The system targets microglia to inhibit Wnt5a expression, resulting in long-lasting analgesic effects. Reprinted with permission from Ref. [104]. Copyright BioMed Central 2024.

In addition to carrying analgesic chemical drugs, MSNs have also been used to carry various biomolecules, including DNA, siRNA, mRNA, and CRISPR-based gene editing tools. For instance, Tao's team engineered an MSN delivery system by loading miR-26a-5p onto surface-aminated MSNs *via* electrostatic adsorption (Fig. 5) [104]. This approach shielded miR-26a-5p from rapid degradation and premature release. Mechanistically, miR-26a-5p exerted analgesic effects by inhibiting Wnt5a. This suppressed the noncanonical Wnt5a/CaMKII/NFAT pathway, thereby reducing microglial activation and the subsequent release of proinflammatory cytokines (IL-1β, IL-6, TNF-α). By further functionalizing the nanoparticle surface with the MG1 targeting peptide, the system achieved precise microglial uptake, significantly amplifying analgesic potency and extending therapeutic efficacy to 6-7 weeks in SNI mice.

As introduced, MSNs possess prominent porous structure, high physiological stability, facile surface modification capability, and mature synthetic procedures, making them widely investigated as carriers for drug and gene delivery. Nevertheless, their extremely slow *in vivo* biodegradability tends to cause irreversible accumulation in the spleen and neural tissues, which becomes the major obstacle to practical clinical translation. Consequently, no MSN-based therapeutics have yet been approved for clinical use, as persistent biosafety concerns continue to limit their broader biomedical application.

#### 3.1.5 Injectable nano-composite hydrogels

Injectable hydrogels are biomaterials capable of transitioning from a liquid state during administration to a robust three-dimensional network post-injection. Due to their capacity for controlled release, targeted delivery, and tunable mechanical properties, they have emerged as a cornerstone of modern drug delivery systems. Their therapeutic efficacy is defined by three core characteristics: First, superior injectability and *in situ* molding. These materials remain fluid *in vitro*, enabling minimally invasive delivery, and undergo rapid gelation *in vivo* in response to physiological stimuli. For example, Lu et al. developed a multifunctional hydrogel (DAGQD@Cu@KGN SAN) for cartilage repair in RA [105]. By leveraging high-efficiency enzyme-like ROS scavenging, this system effectively neutralized the inflammatory environment of the joint cavity. Notably, it remained stable for over 28 days despite frequent joint movement, overcoming the common challenge of material displacement or diffusion seen in traditional hydrogels.

**Fig. 6:**
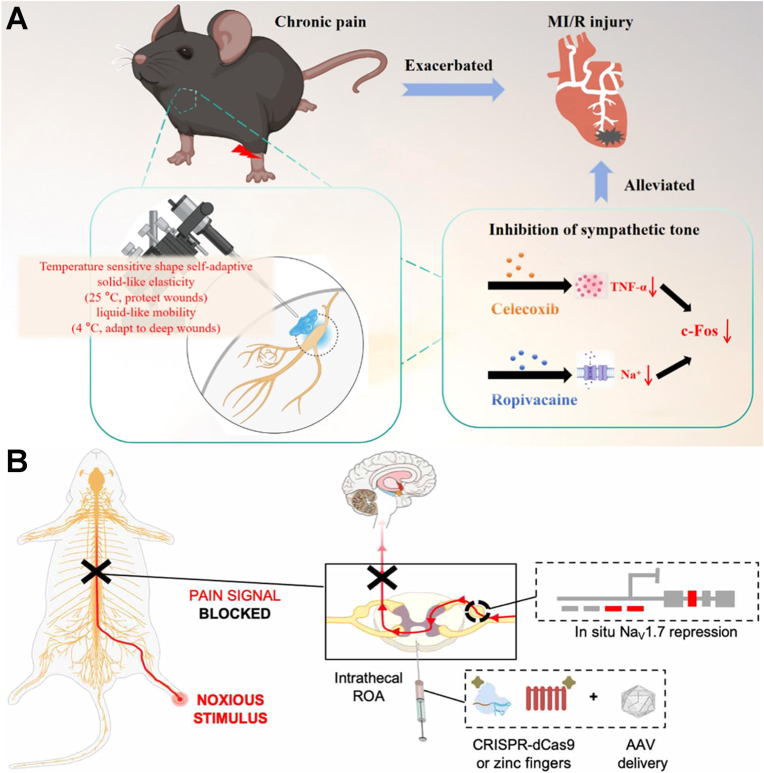
(A) Schematic diagram of synthesis and treating chronic pain-exacerbated MI/R injury of injectable Pluronic/alginate-based composite hydrogel loaded with celecoxib and ropivacaine. Reprinted with permission from Ref. [106]. Copyright Elsevier 2025. (B) Schematic representation of the strategy used for *in situ* Na_V_1.7 repression using ZFP-KRAB and KRAB-dCas9 *via* the intrathecal route of administration. Reprinted with permission from Ref. [113]. Copyright American Association for the Advancement of Science 2021.

Second, high drug loading and sustained release. Nanocomposite hydrogels can incorporate various analgesics through physical encapsulation, chemical bonding, or electrostatic adsorption. For instance, Xie's group developed an injectable, thermosensitive Pluronic/alginate/Laponite-dopamine hydrogel (CLDAFR) co-loaded with ropivacaine and celecoxib to treat chronic pain-exacerbated myocardial ischemia-reperfusion (MI/R) injury [106]. This system transitions from a 4 °C sol-state to a 37 °C gel-state *in situ*, providing biocompatible, adhesive, and sustained drug release. By targeting the superior cervical ganglion (SCG), CLDAFR suppressed neuronal hyperactivity and downregulated TNF-α and c-Fos (Fig. 6A). In CCI mouse models, this localized modulation of the SCG-cardiac sympathetic circuit significantly reduced pain hypersensitivity and myocardial infarct size, offering a targeted strategy for pain-related cardiovascular complications. Recent advancements have also integrated targeted nanocarriers within the hydrogel matrix. Zhang et al. developed a supramolecular system (PNSH@MTX) by cross-linking pullulan with methotrexate-loaded NPs *via* a Schiff base reaction [107]. The nanoparticle surface was modified with oxidized pullulan to precisely target macrophages *via* mannose receptor recognition, effectively reshaping the inflammatory microenvironment to arrest RA progression.

Third, pain signal modulation and microenvironment targeting. Beyond delivery, these hydrogels can actively mitigate pain by modulating pathological signaling. Jiao's group designed a hydrogel that cleared extracellular RNA while releasing bevacizumab to block neurovascular formation at the bone-cartilage interface [108]. By utilizing positively charged components to neutralize negatively charged extracellular RNA, the hydrogel disrupted the recruitment of pro-neurovascularization factors (such as VEGF). This suppresses abnormal signaling and inhibits neurovascularization, treating OA pain at its biological source. As introduced, injectable nanocomposite hydrogels offer significant advantages, primarily due to their ability to undergo in-situ gelation *via* minimally invasive administration. This allows for prolonged residence at the pain site and sustained drug release, effectively reducing dosing frequency. Their extracellular matrix-mimetic structure ensures excellent biocompatibility and enables the co-loading of small molecules and nanoparticles for synergistic analgesic and regenerative effects. However, clinical translation remains hindered by several hurdles, including the need to mitigate initial burst release, optimize loading efficiency, and establish the long-term biosafety of the materials.

#### 3.1.6 Viral vector and sEVs

Apart from the synthetic NPs, natural nanocarriers are increasingly being explored for chronic pain management. A primary example is the sEVs. As previously introduced, they are naturally secreted vesicles that can be modified through genetic engineering, biomaterial integration, and drug loading. While their versatility as delivery platforms for chemotherapeutics and nucleic acids is well-established in oncology and regenerative medicine, their application is now expanding into pain modulation [59]. By leveraging their innate transport mechanisms, sEVs can precisely ferry analgesic agents to targeted pathways, thereby minimizing systemic toxicity [73,111]. Ultimately, their superior biocompatibility and circulatory stability represent a transformative shift toward safer, more effective pain interventions. However, low yields, production scalability issues, and inconsistent batch quality complicate their composition and ultimately obstruct standardized clinical translation.

In addition, viral vectors are modified viruses used to deliver genetic material into cells. In pain relief, they represent the frontier of treatment, moving away from temporary pills toward long-term, gene-based biological cures. For the viral carriers, herpes simplex virus type 1 (HSV-1), adeno-associated virus (AAV), adenoviruses et al. are the most frequently used ones of gene therapy for pain relief [[129], [130], [131], [132]]. For instance, HSV-1 has been used to deliver genes that encode pain-relieving proteins, such as enkephalins and endorphins, to the spinal cord and brain [109,110]. AAV, on the other hand, has been used to deliver genes that encode for pain-relieving proteins, such as IL-10, to the spinal cord and peripheral nervous system [112]. In addition, AAV has also been employed to deliver genome engineering tools to achieve targeted epigenetic repression of Na_V_1.7, thereby inducing relief from chronic pain [113]. Na_V_1.7, a subtype of Na_V_, has been identified as a promising target for addressing pathologic pain states and developing chronic pain therapies, as loss-of-function mutations in Na_V_1.7 have been linked to congenital insensitivity to pain. Notably, Mali's group successfully utilized AAV-mediated delivery of clustered regularly interspaced short palindromic repeats-Cas9 (CRISPR-Cas9) and zinc finger protein (ZFP) to block Na_V_1.7 (Fig. 6B), resulting in reduced thermal hyperalgesia in inflammatory pain models and decreased tactile allodynia in neuropathic pain models, while preserving normal motor function in mice. As clinically validated gene delivery vectors, viral carriers benefit from established technical frameworks. Currently, several viral-based formulations for pain management have entered clinical trials, underscoring the potential of gene therapy for chronic pain. However, analgesic efficacy depends heavily on the precise screening and selection of pain targets. This target compatibility is a primary determinant of therapeutic success and clinical translation. Furthermore, concerns regarding immunogenicity and biosafety continue to limit their widespread clinical adoption.

### 3.2 Nanomaterials with intrinsic pain relief function

In addition to serving as a delivery platform, some nanomaterials possess intrinsic analgesic properties. Notably, sEVs are nanoscale biological particles that function as inherent analgesics. They also represent a type of nanomaterial with inherent analgesic effects. Specifically, some studies have demonstrated that stem cell-derived sEVs exert direct anti-inflammatory and pain-relieving effects. Since these properties were detailed in Section 2, they will not be further elaborated upon here. While research in this area is currently limited, we have organized the existing research by mechanism to provide a comprehensive discussion on these emerging materials (Table 2).

**Table 2:** Nanomaterials with intrinsic analgesic properties for chronic pain management.

Types	Nanomaterials	Targets	Pain model	Duration of action	Analgesic effect	Reference
ROS-regulating nanomaterials						
Nanozymes	MnO_2_@TMNP	ROS, c-FOS/GFAP signaling pathway	Discogenic pain model	>7 days	MnO_2_@TMNP reduced CGRP expression in the dorsal root ganglion and downregulated c-FOS, GFAP in spinal cord segments	[133]
	C_60_FAS	ROS	Formalin-induced acute inflammatory pain model	>30 min	C_60_FAS attenuated the reduction of muscle contraction force induced by inflammation	[134]
	CONPs	Macrophage Polarization	Spinal cord injury-induced neuropathic pain model	>7 days	After CONPs treatment, the CD86^+^/ED1^+^ cells were significantly decreased while the CD206^+^/ED1^+^ cells were significantly increased	[135]
	CNPs	ROS, MDA, NO	Chronic Constriction Injury	>14 days	TNF-α, IL-1β, NO, and MDA levels in the spinal cord were significantly decreased, while total thiol content was significantly increased	[136]
	Fe_3_O_4_ NPs	ROS, iNOS, MPO	Inflammatory pain model induced by CFA	>3.5 h	After Fe_3_O_4_ NPs treatment, the expression of CD68 and MPO were reduced	[137]
	SOD&Fe3O4@ZIF-8	ROS, MAPK/p-65 signaling pathway	CFA-induced inflammatory pain	>24 h	SOD&Fe3O4@ZIF-8 relieved CFA-induced mechanical allodynia and reduced TNF-α, IL-6, and IL-1β levels in the spinal cord	[138]
Hydrogen nanogenerator	Si-based agent	ROS, NLRP3–caspase-1–GSDMD pyroptosis pathway	Trigeminal neuralgia model	Up to 28 days	The expression levels of NLRP3, caspase-1, GSDMD, and IL-1β were significantly downregulated.	[139]
	PDAB	ROS, ROS/NF-κB pathway	CFA-induced arthritis	48 days	PDAB + NIR treatment significantly inhibited the expression of IL-1β and TNF-α, as well as the activation of microglia in the spinal cord	[140]
Ion channel-regulating nanomaterials	MNPs-TRPV1	TRPV1 channels	DMM-induced OA	12 weeks	MNPs-TRPV1 with AMF reduced knee pain sensitivity and improved gait and spontaneous ambulatory activity in DMM mice	[141]
	Cit-AuNRs@Anti-TRPV1	TRPV1 channels	DMM-induced OA	12 weeks	Cit-AuNRs@Anti-TRPV1 under NIR increased paw withdrawal thresholds and improved physical activities in DMM mice	[142]
	BIONSs	TRPV1 channels, Ca^2+^ signal	4T1-induced cancer-induced bone pain	>6 h	BIONSs + US increased hind PWT and PWL, downregulated TRPV1, and inhibited WDR neuron activity in CIBP mice	[143]
Multimodal therapeutic nanoplatforms						
Magnesium-related NPs	MgO NPs	N-methyl-D-aspartate receptor	Formalin-induced acute inflammatory pain model	>25 min	The analgesic effect was more significant.	[144]
	MgB_2_ NPs	N-methyl-D-aspartate receptor, Ca^2+^ signal	CFA-induced inflammatory pain, Chemotherapy-induced neuropathic pain, Chronic constriction injury pain	>4 h	The analgesic effect was significantly stronger than MgSO_4_ and morphine, with no drug tolerance.	[145]
ZnO NPs	nZnO	N-methyl-D-aspartate receptor	Rat acute thermal pain model	>1.5 h	The analgesic effect was gradually enhanced with the extension of time at effective doses.	[146]
	ZnO NPs-EM	COX-1/COX-2	Acetic acid-induced writhing test	>1.5 h	ZnO NPs-EM produced significant antinociceptive effects in a dose-dependent manner as early as 30 min.	[147]
\	FR-ZnONPs	N-methyl-D-aspartate receptor	Glutamate-induced nociception test, Capsaicin-induced neurogenic nociception test, Formalin-induced acute inflammatory pain	>2 h	Rapid onset, long-lasting for 2 h and potent analgesia	[148]
	ZnO NPs	N-methyl-D-aspartate receptor	Glutamate-induced nociception test, Capsaicin-induced neurogenic nociception test, Formalin-induced acute inflammatory pain	30 min	ZnO NPs produced significant antinociceptive effects in a dose dependent manner as early as 30 min.	[149]
sEVs	BMSCs-derived exosomes	RANKL-RANK-TRAF6 pathway	LFJ OA mouse model	5 weeks	BMSCs-derived exosomes inhibited the RANKL-RANK-TRAF6 signaling pathway and reduced MMP13 expression	[66]
	huc-MSCs-derived exosomes	TLR2/MyD88/NF-κB pathway	Chronic Constriction Injury	7 days	Exosomes significantly downregulated the expression of Rsad2, TLR2, MyD88, and p-p65 in the spinal cord	[67]
	hPMSCs-derived exosomes	Wnt5a/Ryk/CaMKII/NFAT	Spared Nerve Injury	35 days	miR-26a-5p significantly downregulated the expression of Wnt5a, Ryk, CaMKII, and NFAT	[68]
	UCMSC-derived exosomes	CD44, c-Fos	Spinal Nerve Ligation	7 days	Exosome treatment significantly suppressed the upregulation of TNF-α and IL-1β, and enhanced the expression of IL-10, BDNF, and GDNF in the ipsilateral L5/6 DRG	[69]
	iPSC-derived exosomes (iPSC-Exos)	PI3K-AKT pathway	Rat sciatic nerve crush injury model	28 days	iPSC-Exos significantly upregulated the protein expression of IL-10, BDNF, S100β, PMP22, and MPZ in the injured sciatic nerve	[70]
	Hypo-VSC EVs	PIK3R2, PI3K/Akt-NF-κB pathway	IR-induced pain	Up to 72 h	Hypo-VSC EVs significantly downregulated PIK3R2, activated the PI3K/Akt pathway, and markedly inhibited the phosphorylation of NF-κB p65 in the spinal cord	[73]

#### 3.2.1 ROS-regulating nanomaterials

Oxidative stress, arising from the imbalance between ROS and antioxidant defenses, is a primary driver of chronic pain pathogenesis. Excessive accumulation of ROS, including superoxide anions (O_2_^•−^), hydroxyl radicals (•OH), and hydrogen peroxide (H_2_O_2_), damages essential cellular components and serves as a critical signaling mediator in pain initiation and chronicity. By activating related pathways, such as mitogen-activated protein kinase (MAPK) and nuclear factor kappa B (NF-κB) pathways, excessive ROS trigger neuroinflammation and the subsequent release of pro-inflammatory cytokines like TNF-α, IL-1β, and IL-6 [150,151]. Furthermore, ROS directly sensitize nociceptive neurons by modulating TRPV1, TRPA1, and Na_V_ channels, effectively lowering pain thresholds and inducing mechanical allodynia [152]. Within the spinal dorsal horn, ROS-driven glial hyperactivation facilitates central sensitization and synaptic plasticity, thereby amplifying pain signals [153]. Such oxidative mechanism is implicated in diverse chronic pain conditions, such as neuropathic pain, inflammatory pain, migraine, postoperative chronic pain, and complex regional pain syndrome [154]. Consequently, targeting ROS regulation represents a promising therapeutic strategy for managing intractable chronic pain.

##### 3.2.1.1 Nanozymes

Nanozymes, nanomaterials engineered to mimic natural catalytic activity, offer a robust and stable alternative to their biological counterparts [155]. By exhibiting antioxidant enzyme-like activities, mainly superoxide dismutase (SOD) and catalase (CAT), nanozymes have emerged as a prominent tool for scavenging excessive ROS and mitigating the oxidative stress underlying chronic pain [126,133,134,156]. One such nanozyme, cerium oxide nanoparticle (CeO_2_), has been studied as a promising candidate for alleviating neuropathic pain [135]. Due to the reversible switching between its Ce^3+^ and Ce^4+^ oxidation states, CeO_2_ exhibits SOD and CAT-like activities using O_2_^•−^ and H_2_O_2_ as substrates, respectively. Forouzanfar's group used pullulan-capped CeO_2_ NPs to manage neuropathic pain in a rat CCI model [136]. Following intravenous administration, CeO_2_ significantly downregulated neuroinflammatory biomarkers, including TNF-α, IL-1β, MDA, and NO and restored thiol levels in the CCI rats. Consequently, the treated rats demonstrated marked improvements in thermal hyperalgesia and allodynia compared to the untreated CCI group.

**Fig. 7:**
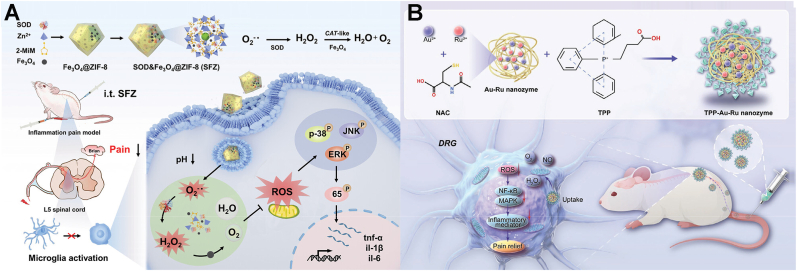
(A) The synthetic process of SOD&Fe_3_O_4_@ZIF-8 NPs with multi-enzyme mimicking activities and its analgesic mechanism on inflammatory pain. Reprinted with permission from Ref. [138]. Copyright Wiley 2023. (B) The preparation of TPP-Au-Ru nanozyme and its underlying mechanism of neuropathic pain relief. Reprinted with permission from Ref. [157]. Copyright Wiley 2024.

In addition, iron oxide (Fe_3_O_4_) NPs have also been explored in analgesia. By mimicking SOD and CAT activity, Fe_3_O_4_ nanozymes scavenged O_2_^•−^ and H_2_O_2_. This reduction in oxidative stress attenuated inflammatory cell infiltration and pro-inflammatory signaling, effectively alleviating chronic inflammatory and neuropathic pain [137,156]. More recently, Shi's group developed a nanocomposite consisting of natural SOD enzyme and Fe_3_O_4_ nanozyme encapsulated in zeolitic imidazolate framework (ZIF)-8 [138]. This pH-responsive nanocomposite selectively targeted inflammatory environments, exhibiting enhanced antioxidative activities and improved analgesic efficacy in a complete Freund's adjuvant (CFA)-induced inflammatory pain mice model (Fig. 7A). Furthermore, the research revealed that the nanocomposite inhibits the activation of MAPK/p-65 signaling pathway, leading to reduced phosphorylated protein levels (p-JNK, p-p65, p-p38, and p-ERK) and inflammatory factors, thereby preventing microglia and astrocyte activation and achieving analgesia.

Furthermore, ruthenium (Ru)-based nanozymes have also been studied in managing chronic neuropathic pain. For instance, Jiang's group researched the function of metal-organic framework-confined Ru nanozyme in treating central post-stroke pain (CPSP). By encapsulating the nanozyme in a p-dodecylbenzene sulfonamide (p-DBSN) modified liposome, the nanocomplex acquired endoplasmic reticulum-targeting capabilities. The obtained MOF nanozymes demonstrated SOD and CAT-like activities to scavenge O_2_^•−^ and decompose H_2_O_2_. Following intravenous administration in CPSP mice, MOF-based nanozyme successfully crossed the blood-brain barrier, accumulating on neuronal endoplasmic reticulum, where it inhibited oxidative stress, reduced matrix metalloproteinases (MMPs) expression, and suppressed neuroinflammation, ultimately leading to effective CPSP treatment [158]. Similarly, Gu's group developed a Ru-contained bimetallic cluster nanozyme (TPP-Au-Ru) with mitochondria-targeting ability and explored its impact on neuropathic pain [157]. The nanozyme demonstrated pain relief in a mouse model of CCI by scavenging ROS (such as O_2_^•−^ and H_2_O_2_), reducing inflammation, and protecting mitochondrial function through inhibition of the MAPK and NF-κB signaling cascades (Fig. 7B). Notably, a single intravenous injection of TPP-Au-Ru yielded a 36-h analgesic effect with minimal biological toxicity.

##### 3.2.1.2 Hydrogen nanogenerator

Beyond nanozymes, hydrogen-generating nanomaterials represent a distinct class of ROS scavengers. Hydrogen is a potent therapeutic gas characterized by exceptional biosafety, high tissue permeability, and selective free radical scavenging capabilities (•OH and ONOO^−^) [159]. It has been investigated across various pathologies, notably for managing inflammatory symptoms and associated pain [160,161]. Based on the mechanism, Yu's group evaluated the therapeutic potential of nano-silicon for treating trigeminal neuralgia (TN). The pathogenesis of TN is primarily characterized by trigeminal ganglion demyelination, driven largely by chronic neuroinflammation. Hydrogen has been shown to downregulate the NOD-like receptor family pyrin domain containing 3 (NLRP3) inflammasome by scavenging mitochondrial ROS [162]. When administered *via* oral gavage, nano-silicon acted as a safe, sustained intestinal source of hydrogen, leaving only non-toxic SiO_2_ as a byproduct. Experimental data indicated that nano-silicon inhibited microglial pyroptosis by blocking the NLRP3-caspase-1-Gasdermin D pathway, thereby reducing demyelination and alleviating pain in TN rat models [139]. Furthermore, Chen's group developed aminoborane-modified polydopamine nanospheres (PDAB) as a responsive hydrogen nanogenerator [140]. Utilizing aminoborane as a precursor, PDAB ensured a stable hydrogen supply triggered by the mildly acidic RA microenvironment (pH 4.7-6.8), an effect further enhanced under near-infrared (NIR) irradiation. In a CFA-induced RA mouse model, the PDAB + NIR strategy slowed disease progression by reducing chondrocyte apoptosis and inhibiting pathological angiogenesis. Furthermore, behavioral assessments and molecular analyses indicated that it alleviated RA pain by suppressing central sensitization *via* the ROS/NF-κB pathway.

#### **3.2.2** **Ion channel**-**regulating nanomaterials**

As previously noted, ion channels play a key role in pain processing. They are considered a major class of drug targets for modulating sensation and controlling chronic pain. Recently, certain nanomaterials have also demonstrated the ability to regulate ion channels and are being explored for chronic pain management. Notably, TRPV1 plays a major role in nociception and neurogenic inflammation. It is primarily expressed in sensory neurons, particularly those involved in nociception (the detection of painful stimuli). TRPV1 is activated by intense heat, low pH, capsaicin, and specific chemicals. This activation triggers a non-selective influx of positively charged ions, primarily calcium and sodium, into the cell. Consequently, the neuron undergoes depolarization and discharges action potentials, which transmit pain signals to the brain [163]. For instance, in the case of tissue inflammation or nerve injury, TRPV1 can undergo sensitization under the stimulation of local inflammatory mediators, such as bradykinin, prostaglandin E2, nerve growth factor and adenosine triphosphate. This sensitization is characterized by a decreased activation threshold, increased channel current, and enhanced membrane localization. Concurrent exposure to multiple stimuli amplifies TRPV1 activation. However, prolonged stimulation eventually diminishes neuronal responsiveness. This leads to a gradual or total loss of sensitivity, inducing a stimulus-specific habituation known as tachyphylaxis [164]. Based on it, researchers have explored its therapeutic potential by investigating the efficacy of agonists and antagonists to alleviate pain [[165], [166], [167], [168]]. Notably, the therapeutic use of TRPV1 modulators depends on the specific pathophysiological drivers of chronic pain. TRPV1 antagonism is primarily indicated for conditions involving receptor upregulation and hypersensitivity, such as inflammatory, neuropathic, and nociplastic pain. In these contexts, antagonists elevate the nociceptive threshold and suppress ectopic signaling. Conversely, TRPV1 agonism leverages receptor exhaustion. Low-dose agonists achieve analgesia through acute desensitization and potential neuroregeneration (e.g., post-injury pain), while high-dose agonism induces terminal defunctionalization by retracting nociceptor terminals (such as OA pain) [169]. Ultimately, the therapeutic choice depends on whether the goal is functional blockade or structural silencing *via* overstimulation. In addition, reducing TRPV1 expression or membrane receptor density *via* targeted siRNA offers another therapeutic pathway, especially for chronic pain conditions involving substantial TRPV1 upregulation and neuronal hypersensitivity [36].

**Fig. 8:**
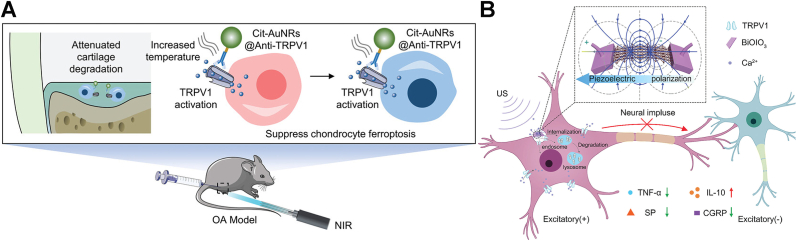
(A) Schematic illustration of Cit-AuNRs@Anti-TRPV1 switch for photothermal activation of TRPV1 signaling to attenuate OA. Reprinted with permission from Ref. [142]. Copyright Wiley 2024. (B) Schematic diagram of ultrasound triggered piezoelectric BiOIO_3_ nanosheets for cancer-induced bone pain relief. Reprinted with permission from Ref. [143]. Copyright Wiley 2024.

Recently, nanomaterials have also been employed to modulate TRPV1 expression for pain relief in various models. For instance, research has shown that the activation of TRPV1 significantly alleviates OA pain [141,170,171]. Clinical trials have explored intra-articular injection of capsaicin (the agonist of TRPV1) and topical application of capsaicin cream, but these methods have limitations, including short-lived TRPV1 activation, skin damage, and unavoidable toxicity, highlighting the need for more controlled, effective, and targeted approaches. To resolve it, Shi's group developed a TRPV1-targeting approach using monoclonal antibody-conjugated citrate-stabilized gold nanorods (Cit-AuNRs@Anti-TRPV1) to selectively activate TRPV1 thermally (Fig. 8A). Intra-articular injection of Cit-AuNRs@Anti-TRPV1, followed by NIR irradiation, effectively activated TRPV1. Such persistent activation not only induced desensitization of TRPV1-expressing sensory neurons, but also suppressed chondrocyte ferroptosis, and attenuated cartilage degradation, thereby alleviating osteophyte formation, subchondral bone sclerosis, and pain in destabilization of the medial meniscus-induced OA mice, ultimately improving their physical activities [142].

Furthermore, piezoelectric materials have emerged as a novel approach for managing chronic pain through reducing TRPV1 membrane receptor density. For instance, Bu's group developed piezoelectric BiOIO_3_ nanosheets (BIONSs) and evaluated their mechanical and thermal pain relief efficiency in a cancer-induced bone pain (CIBP) mouse model [143]. Following intrathecal injection of BIONSs into mice, ultrasound application generated a surface electric field that induced TRPV1 internalization (Fig. 8B). Although the precise mechanism underlying this piezoelectric-induced internalization remains to be fully elucidated, the resulting downregulation of TRPV1 expression reduced Ca^2+^ signaling in spinal neurons and inhibited activity of wide dynamic range neurons, ultimately preventing CIBP transmission. Consequently, CIBP relief was achieved for up to 6 h with 4-min ultrasonication.

#### 3.2.3 Multimodal therapeutic nanoplatforms

While the previously discussed nanomaterials address pain by targeting either ROS homeostasis or ion channel activity, others possess an inherent ability to engage multiple pathways simultaneously. This section examines these multi-mechanism nanomaterials and their concurrent modulation of ROS, ion channels, and inflammatory signaling.

##### 3.2.3.1 Magnesium-related NPs

Magnesium sulfate acts as an NMDA receptor antagonist and a physiological calcium channel blocker [172]. NMDA receptors are excitatory ionotropic glutamate receptors that regulate synaptic transmission and plasticity by mediating calcium influx. As these receptors are critical targets in neuroexcitotoxicity and central sensitization, magnesium sulfate provides antinociceptive and analgesic benefits. However, since its effects are limited when used alone, it is primarily used as an adjuvant to enhance other analgesics [173]. By development of nanotechnology, magnesium oxide (MgO) NPs, a new kind of magnesium supplements, have been studied with analgesic and anti-inflammatory effects. The effect might due to the high permeability effect of MgO NPs in the central nervous system [144]. Recently, Bu's group prepared MgB_2_ nanosheets with enhanced analgesic efficiency than magnesium ions [145]. Since under physiological conditions, the nanosheet underwent hydrogenation, generating 2D borophene-like BH. The generated BH had the ability to attract the extracellular Na^+^ and K^+^ through cation-pi interactions. This effect led to an alteration in the ion distribution at the cellar membrane for a decrease in sodium currents and an increase in potassium currents in neurons. In coordination with the released Mg^2+^ to block calcium channels, MgB_2_ effectively raised the rheobase and suppresses the action potential firing. Furthermore, the electron-rich boron endowed MgB_2_ with dose- and pH-dependent antioxidative properties, enabling the scavenging of •OH, H_2_O_2_ and O_2_^•−^ to provide neuroprotection. The synergistic works of MgB_2_ provided a potent analgesic effect, superior to both MgSO_4_ and morphine for clinical use.

##### 3.2.3.2 ZnO NPs

Zinc, a ubiquitous trace element in the animal body, has been shown to modulate pain sensation when administered exogenously. In rodents, intraperitoneal administration of ZnCl_2_ has been found to alleviate thermal hyperalgesia and neuropathic pain [174], while intrathecal administration of a zinc chelator induces thermal hyperalgesia [175]. Zinc's analgesic effects are primarily attributed to its anti-inflammatory properties and inhibition of NMDA receptor activity, which reduces pain transmission and modulation. Moreover, zinc enhances its pain-relieving effects by elevating gamma-aminobutyric acid levels, an inhibitory neurotransmitter, and subsequently reducing glutamate release [176]. Furthermore, intracellular zinc's inhibition of TRPV1 receptors further contributes to its analgesic properties [177]. More recently, Lee's group discovered that metallothionein-3-mediated intracellular zinc triggers antioxidant and anti-inflammatory responses in a mouse model of inflammatory pain induced by CFA [178]. Zinc oxide (ZnO), a zinc supplement, can gradually release zinc ions in biological environments. Unlike conventional forms, nanomedicine offers the advantages of reduced dosage and side effects, making it an attractive opportunity. By harnessing this potential, Torabi's group explored the analgesic effects of nano ZnO (nZnO), a promising alternative. Their results showed that nZnO exhibited a higher analgesic effect than its conventional counterpart in the hot plate assay using Wistar rats [146]. Subsequently, various green route methods were employed to synthesize nZnO, all of which exhibited analgesic activity [147,148]. Notably, Wang's group synthesized nZnO from *Vernonia amygdalina* and evaluated their analgesic potential in murine models. Through acetic acid-induced writhing, glutamate, capsaicin, and formalin tests, nZnO exhibited dose-dependent antinociceptive activity. Mechanistically, these effects were attributed to the inhibition of TRPV1 receptors, blockade of glutamate-mediated pain pathways, and reduction of prostaglandin synthesis. Furthermore, nZnO significantly attenuated leukocyte infiltration and downregulated pro-inflammatory cytokines, including TNF-α, IL-1β, and IL-6. Notably, these analgesic and anti-inflammatory effects occurred without impairing locomotor activity, supporting the therapeutic promise of green-synthesized nZnO [149].

## 4 The targeted therapy strategies

A key hallmark of nanomaterials is that they can be readily functionalized to achieve targeted delivery, which minimizes systemic side effects while maximizing analgesic efficacy. Modifying nanomaterials with specific ligands to engage pain-signaling molecular, cells or tissues constitutes an active targeting strategy. Furthermore, endowing nanomaterials with stimulus-responsive capabilities represents another targeting approach. This responsive release/activation strategy has evolved into two paradigms: endogenous stimuli, which leverage the biological microenvironment, and exogenous stimuli, which utilize external triggers, such as light, ultrasound, or magnetic fields, to control the timing and location of drug release.

### 4.1 Active targeting strategy

Chronic pain presents a diverse array of therapeutic targets, which can be broadly categorized into signaling proteins (e.g., Nav1.7, TRPV1, and inflammatory cytokines), cellular mediators (such as sensory neurons, microglia, astrocytes, and infiltrated immune cells), and regional sites (including the DRG, spinal dorsal horn, and inflamed peripheral sites) [179,180]. To engage these targets, NPs have been engineered with specific ligands, demonstrating significant therapeutic potential.

#### 4.1.1 Targeting signaling proteins

The pathogenesis of chronic pain involves a complex network of signaling proteins. Ligand-modified NPs provide a targeted therapeutic strategy by specifically binding to these proteins and modulating pain-related signaling cascades. For instance, as mentioned above, monoclonal antibody-conjugated gold nanorods (Cit-AuNRs@Anti-TRPV1) can selectively activate TRPV1 receptors *via* photothermal effect, inducing OA pain alleviation [141]. In addition to ion channel proteins, the cytokine family also serves as pivotal targets for nanocarrier-based active targeting. Cytokines are small, secreted proteins that mediate cellular communication and inflammatory signaling. They function through autocrine (acting on the secreting cell), paracrine (acting on nearby cells), or endocrine (acting on distant cells) pathways. As cytokines are broadly categorized as pro- or anti-inflammatory, neutralizing pro-inflammatory cytokines and/or increasing expression of anti-inflammatory cytokines is vital for managing inflammatory disorders and associated pain [181]. Although antibody-based therapies have revolutionized treatment due to their clinical efficacy, they possess significant limitations. These include short half-lives that diminish therapeutic impact and severe systemic side effects, such as increased risks of infection, malignancy, or infusion reactions. Furthermore, because cytokines are expressed ubiquitously, systemic antibody administration often interferes with non-target biological mechanisms. To address these challenges, Neves's group developed chitosan-hyaluronic acid NPs functionalized with surface-bound anti-TNF-ɑ and anti-IL-6 antibodies [182]. This platform extended the antibodies' therapeutic window by protecting them from degradation and minimized off-target effects through local administration. These biofunctionalized NPs successfully mitigated the harmful effects of inflammatory macrophages on human chondrocytes, demonstrating a synergistic dual neutralization effect. Notably, in an *in vivo* inflammatory model, these NPs ameliorated inflammation and pain more effectively than soluble antibodies, exhibiting a superior safety profile and prolonged bioactivity.

However, because cytokine networks are often redundant and compensatory, current techniques targeting only a subset of cytokines frequently fall short. Cell membrane-coated NPs offer a promising solution by replicating the complex cellular interactions observed in disease pathology, enabling multiplex cytokine scavenging. Zhang's group has researched this extensively, utilizing membranes from neutrophils, macrophages, and cancer cells to sequester cytokines in chronic inflammatory disorders [[183], [184], [185]]. While their impact on pain relief remains unexplored, these strategies show significant potential for treating inflammation-driven diseases.

#### 4.1.2 Targeting pain-related cells

The onset and regulation of pain involve multiple cell types in the body. Glia, especially astrocytes and microglia have been recognized as powerful modulators of pain and have emerged as targets for drug development [186]. Although glia is well known for having a number of housekeeping functions that are necessary for healthy neuronal communication, on strong activation they act as immune-responsive cells or exert a neuroprotective effect. Glia can contribute to neuropathic-pain processing by releasing a number of glial and neuronal signaling molecules. There is substantial evidence that both astrocyte and microglia activation lead to pro-inflammatory responses with pathological effects, such as neuronal hyperexcitability, neurotoxicity and chronic inflammation. Thus, targeted anti-inflammation and anti-oxidation in glia show promise for pain relief. There have been several strategies for realizing glia targeting. One is using peptide. The MG1 peptide has been found to have a high affinity for M1 microglia, which has been demonstrated to guide NPs into microglia cells and enhance the therapeutic effect in autism spectrum disorder, neuroinflammation and chronic pain [104,187].

Another effective strategy involves antibody-based surface modification. Antibodies or antibody fragments can enable highly precise targeting of NPs by recognizing specific antigens or receptors expressed on the surface of microglia. For example, lipid NPs modified with anti-CD11b antibodies and loaded with the anti-inflammatory agent curcumin have been shown to selectively bind to activated microglia within the spinal dorsal horn [188]. Once internalized *via* cellular uptake, the curcumin was released to suppress pro-inflammatory signaling, effectively reducing the expression of key cytokines such as TNF-α and IL-6. This targeted approach significantly elevated both mechanical and thermal pain thresholds, alleviating neuropathic pain, while demonstrating markedly lower systemic toxicity than non-modified formulations. In addition, some literature also indicated curcumin could switch microglial phenotypes from M1 to M2 by suppressing astrocytic activation, reducing proinflammatory cytokine release, promoting anti-inflammatory cytokine production, and contributing to relief of neuropathic pain [189].

**Fig. 9:**
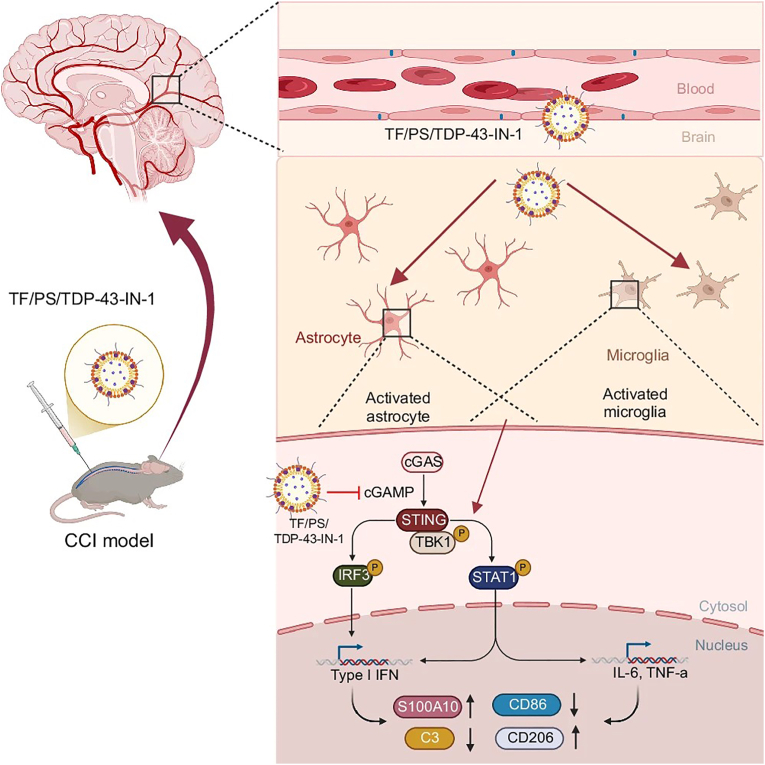
Schematic illustration of the molecular mechanism of TF/PS/TDP-43-IN-1 regulating the cGAS-STING inflammatory pathway, mediating microglia-astrocyte crosstalk and affecting neuropathic pain. Reprinted with permission from Ref. [190]. Copyright Springer Nature 2025.

More recently, Miao's group developed liposomes modified with transferrin (TF) and phosphatidylserine (PS) to deliver a TDP-43 aggregation inhibitor (TF/PS/TDP-43-IN-1) [190]. This delivery system effectively crossed the blood-brain barrier *via* TF-mediated transport and targeted glial cells through PS signaling (Fig. 9). TDP-43 is a nuclear RNA/DNA binding protein essential for RNA splicing. However, under pathological conditions, it mislocalized to the cytoplasm and formed aggregates, a key upstream trigger for neuroinflammation and neuronal dysfunction. By inhibiting these aggregates, the TF/PS/TDP-43-IN-1 complex modulated the cGAS-STING inflammatory pathway, reducing the pathological crosstalk between microglia and astrocytes. Ultimately, this targeted approach significantly alleviated neuropathic pain by suppressing the neuroinflammatory processes driven by glial activation.

Furthermore, nanoparticle delivery can also exploit intrinsic physicochemical properties, such as particle size, surface charge, and hydrophobicity, to take advantage of the altered CNS microenvironment during chronic pain. By optimizing these factors, researchers can achieve preferential accumulation within microglia without complex surface engineering. For instance, curcumin-loaded PLGA NPs with a diameter of approximately 150 nm were efficiently internalized by microglia, leading to the suppression of pro-inflammatory activation and the attenuation of neuropathic pain [191]. Beyond small molecules, PLGA has also been successfully used to deliver anti-inflammatory genes directly into mouse spinal cord cells, specifically microglia [192]. In models of sciatic nerve transection, this microglial-specific gene delivery produced significant analgesic effects, highlighting the versatility of PLGA as a vehicle for both chemical and genetic pain therapies.

Despite the promise of glia-targeted nanomedicine, several significant hurdles hinder its clinical translation. A primary challenge is the blood-spinal cord barrier, which remains highly selective and limits the penetration of systemically injected NPs into the spinal dorsal horn. Even when chronic inflammation partially compromises this barrier, achieving consistent, therapeutic drug concentrations specifically within glial cells remains difficult. Furthermore, the heterogeneity of glial phenotypes poses a major biological hurdle. Glia cells exist along a complex spectrum ranging from pro-inflammatory to neuroprotective states rather than a simple binary on/off switch. Indiscriminately suppressing these cells risks disrupting their essential homeostatic functions. The temporal window for intervention is also narrow and poorly defined, as glial activation typically peaks during the early transition from acute to chronic pain. Finally, while the current literature focuses heavily on microglial targets, the specific roles and targeting strategies for astrocytes and other glia remain largely unexplored, representing a significant gap in our understanding of glial-mediated pain relief.

#### 4.1.3 Targeting regional sites

Strategically targeting pain-related tissues, such as peripheral injury sites, the spinal cord, and dorsal root ganglia, is essential for the precise management of chronic pain. While local injections provide site-specific delivery, they remain highly operator-dependent and difficult to standardize for clinical use. Consequently, developing nanomaterials with active tissue targeting capabilities is vital to ensure selective enrichment at pathological sites, thereby enhancing therapeutic efficacy while minimizing off-target effects. Among them, DRG serves as a critical clinical target for pain management because it functions as a primary gateway for sensory transduction and pain modulation. Despite only being the size of a peanut, each ganglion houses up to 15,000 neurons and is encased within rigid bony structures that leave no room for expansion. This anatomical confinement makes the DRG highly susceptible to mechanical compression and inflammation from conditions like herniated discs or osteophytes [193]. Furthermore, peripheral injuries can trigger a chronic cycle of inflammation within the DRG. Supportive glial cells release immune markers and neurotropic factors that cause the glia to grow, physically compressing the sensory neurons. This creates a persistent feedback loop of pain that often continues even after the original site of injury has healed. By targeting the DRG directly through modern methods, like neuromodulation or radiofrequency, clinicians can interrupt these signals at their source, offering a more precise and effective solution for chronic neuropathic pain than traditional systemic treatments [194].

**Fig. 10:**
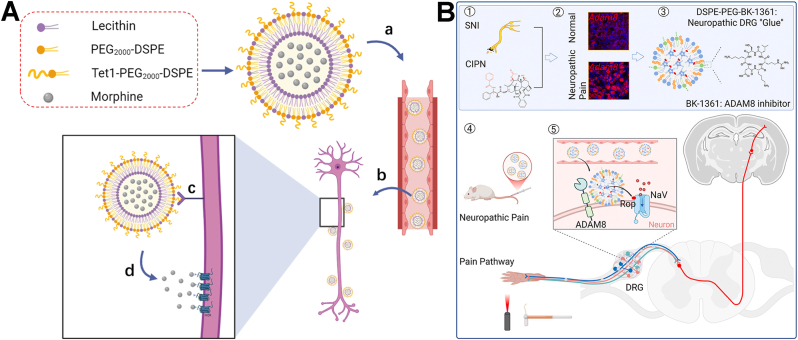
(A) Schematic illustration of the formulation of the peptide-decorated lipid nanoparticle Tet1-LNP (morphine) and used for systemic morphine delivery and targeted pain treatment. Reprinted with permission from Ref. [198]. Copyright Dove Medical Press 2024. (B) Schematic illustration of BK-1361 conjugated lipid NPs for DRG-targeted ropivacaine delivery to effective relief of neuropathic pain. Reprinted with permission from Ref. [199]. Copyright Elsevier 2025.

Building on its role as a key sensory hub, the DRG has emerged as a primary target for drug delivery. While traditional pharmacological strategies, such as direct, intrathecal, or epidural injections, can modulate DRG ion channels and signaling pathways, these methods are technically demanding and largely confined to mechanistic studies [[195], [196], [197]]. Recently, DRG-targeted nanomedicines have gained significant attention for their ability to deliver conventional analgesics *via* systemic injection. For example, Cao's group developed liposome NPs modified with a Tet1 peptide to encapsulate morphine (Fig. 10A). These NPs were engineered to be larger than free morphine molecules, which restricted their ability to passively diffuse into the brain. Instead, the Tet1 peptide's high affinity for trisialoganglioside receptors on DRG neurons allowed for the selective activation of peripheral opioid receptors, maintaining effective analgesia while eliminating central side effects [198].

Furthermore, Chen's group utilized LNPs modified with an inhibitory peptide targeting a disintegrin and metalloproteinase protein 8 (ADAM8), a membrane-anchored protein typically recognized as a cancer biomarker (Fig. 10B) [199]. They discovered that ADAM8 was persistently upregulated in DRG neurons across two distinct neuropathic pain models: spared nerve injury and chemotherapy-induced neuropathy. When injected intravenously, these LNPs facilitated targeted analgesic accumulation within the DRG. This approach significantly enhanced the intensity and duration of pain relief in both single and repeated treatments. Ultimately, this targeted delivery not only relieved pain but also addressed associated psychiatric comorbidities without triggering latent systemic toxicities, marking a more precise and safer frontier for chronic pain therapy.

Despite its potential, DRG-targeted therapy is hindered by the anatomical challenge of accessing a target encased in rigid bone and the pharmacological difficulty of maintaining drug concentrations against rapid vascular washout. Additionally, the biological complexity of chronic pain, driven by redundant neural pathways and persistent glial-mediated neuroinflammation, often requires multifaceted interventions rather than single-target approaches. While emerging nanomedicines aim to overcome these barriers, ensuring long-term biocompatibility and precise specificity for injured versus healthy ganglia remains a significant hurdle for clinical translation.

### 4.2 Stimulus-responsive release/activation strategies

Active targeting enables precise receptor recognition *via* ligand modification, though it involves arduous screening and potential immunogenicity. Additionally, stimulus-responsive strategies represent another promising approach to chronic pain by enabling site-specific drug release or intelligent nanoparticle activation at the lesion.

#### 4.2.1 Endogenous stimulus-responsive strategy

By utilizing endogenous signals as functional triggers, nanomaterials can achieve high spatial precision in pain management. These systems respond to internal stimuli, such as pH gradients and enzymatic activity, ensuring that the active components are only released or activated upon exposure to the unique molecular signatures of the painful microenvironment. This section explores the various endogenous targeting factors currently being investigated.

##### 4.2.1.1 pH

Pain-associated pathologies, including inflammatory tissues, degenerative joints, and injury sites, are frequently characterized by accelerated metabolism, hypoxia, and the accumulation of lactic acid, which collectively establishes a weakly acidic microenvironment. This localized drop in pH serves as a precise biological trigger for responsive therapeutic interventions. The efficacy of pH-responsive nanomedicine hinges on acid-triggered activation: maintaining structural integrity and cargo retention during systemic circulation, followed by rapid, site-specific transformation or drug release within the acidic microenvironment of the lesion or intracellular compartments. This targeted approach enhances analgesic and anti-inflammatory potency while significantly mitigating systemic side effects. There are three main types of pH-responsive strategies: the cleavage of acid-sensitive covalent bonds, protonizable polymer-driven assembly/disassembly, and acid-triggered nanosupport decomposition. In the first approach, acid-sensitive bonds undergo accelerated hydrolysis in acidic environments to facilitate precise release [200]. For instance, Ikoma et al. conjugated the local anesthetic derivative QX-OH within mesoporous silica NPs *via* ester linkages [201]. The acidic environment triggered bond cleavage, ensuring efficient drug liberation. While this strategy offers a clear triggering mechanism and tunable release kinetics based on bond stability, its efficacy relies on a distinct pH gradient within the pathological site to avoid premature or incomplete release.

In the second approach, protonatable polymer-driven self-assembly leverages pH-responsive polymers to spontaneously form nanocarriers. This mechanism ensures high drug-loading efficiency through rapid charging while enabling sustained release within acidic or target pH environments. A notable example is the composite system developed by Li et al., which integrated pH-sensitive micelles within a thermosensitive injectable hydrogel [202]. In this dual-stage approach, ropivacaine was first encapsulated in a polyaspartamide-based polymer to form micelles, which were subsequently dispersed into the thermosensitive hydroxypropyl chitin hydrogel to create a secondary diffusion barrier. This architecture significantly extended the duration of sciatic nerve blocks, showcasing the clinical potential of pH-responsive phase-change strategies for long-acting local anesthesia. While this dual mechanism effectively addresses both hydrophobic drug loading and sustained release, its primary limitation lies in the potential for physiological fluctuations to disrupt protonation efficiency, leading to inconsistent responsiveness.

**Fig. 11:**
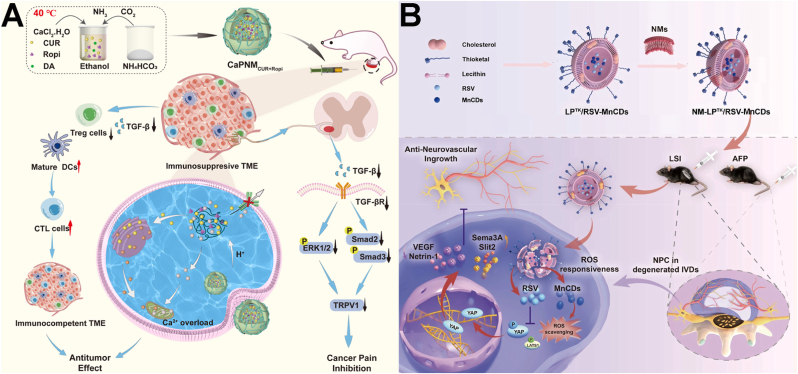
(A) Schematic representation of the preparation process of CaPNM_CUR + Ropi_ and the mechanism for antitumor and cancer pain suppression. Reprinted with permission from Ref. [203]. Copyright America Chemical Society 2024. (B) Schematic illustration of the preparation of biomimetic ROS-responsive NM-LP^TK^/RSV-MnCDs NPs and their therapeutic mechanism for intervertebral disc degeneration. Reprinted with permission from Ref. [204]. Copyright Elsevier 2026.

Thirdly, the disintegration of nanocarrier triggered by acid has also been used for drug release for pain therapy [99]. For instance, Wang's group developed a novel calcium carbonate/polydopamine composite nanoplatform (CaPNMCUR + Ropi) loaded with curcumin and ropivacaine to effectively relieve cancer pain and enhance tumor immunotherapy [203]. In the acidic tumor environment, CaPNMCUR + Ropi degraded, releasing calcium ions, ropivacaine, and curcumin (Fig. 11A). The released calcium ions triggered tumor cell apoptosis, while ropivacaine provided rapid pain relief from tumor injection. Meanwhile, curcumin reduced immunosuppressive and inflammatory factors (TGF-β, IL-6, IL-1β, and TNF-α) in the tumor microenvironment, augmenting the immune response and alleviating inflammatory pain in cancer animals. Additionally, the decrease in TGF-β led to reduced transient receptor potential vanilloid 1 (TRPV1) expression, which is a central molecular integrator of noxious and thermal stimuli and play a major role in nociception and neurogenic inflammation, resulting in long-lasting analgesic effects and alleviated hyperalgesia. In addition, Bu's group developed a smart nanomedicine for the treatment of metastatic bone cancer pain [205]. The nanoplatform utilized Mg/Al layered-double-hydroxide (LDH) nanoshells loaded with the antagonist AZ-23 and surface-modified with alendronate (ALD). By leveraging the bone-targeting affinity of ALD and the pH-responsive degradation of LDH, the system ensured precise delivery and site-specific release of AZ-23 within the acidic tumor microenvironment. Once released, AZ-23 blocked the Nerve growth factor/Tropomyosin receptor kinase A pathway, inhibiting neurogenesis and alleviating pain. Concurrently, the LDH scaffold neutralized excess H^+^, suppressing neural excitation and interrupting pain-mediated Ca^2+^ crosstalk. This synergistic approach reversed Ca^2+^-dependent cell cycle progression, effectively providing simultaneous analgesia and tumor suppression.

Furthermore, in chronic pain, the research indicated the substance P (SP) neurokinin 1 receptor (NK1R) redistributed from the plasma membrane to acidified endosomes, maintaining pain [206]. Thus, targeting NK1R inhibition in endosomes offers a promising approach for pain relief. To this end, Bunnett's group developed pH-responsive NPs by synthesizing diblock copolymers with a hydrophilic shell of P(PEGMA-co-DMAEMA) and hydrophobic cores of P(DIPMA-co-DEGMA), which were then loaded with aprepitant, an NK1R antagonist [207]. Following intrathecal injection into rodents, the NPs accumulated in endosomes *via* clathrin- and dynamin-dependent endocytosis. In response to the acidic environment, the NPs released aprepitant, which inhibited NK1R, thereby preventing SP-induced activation of spinal neurons and pain transmission. Despite extensive research into pH-responsive drug delivery, direct empirical evidence characterizing the acidic microenvironments of key nociceptive tissues, such as the DRG, remains elusive. Consequently, the development of high-resolution sensing techniques to map the interstitial pH of these pain-related tissues represents a critical and promising research frontier.

##### 4.2.1.2 ROS

The pathogenesis of chronic pain is driven by a self-perpetuating cycle of oxidative stress and neuroinflammation, initiated when mitochondrial dysfunction in damaged neurons triggers an overproduction of ROS. These molecules act as critical signaling mediators that activate the NF-κB pathway, leading to a surge of pro-inflammatory cytokines that sensitize peripheral nociceptors and polarize spinal microglia, a hallmark of central sensitization. To address this, ROS-responsive nanomedicine has emerged as a smart therapeutic strategy, utilizing the pathologically elevated ROS levels as a localized biochemical trigger for site-specific activation/drug release. By incorporating ROS-sensitive moieties such as thioketals or boronic esters, these platforms remained stable during systemic circulation but underwent precise disintegration at the pain site (Fig. 11B) [204]. This on-demand delivery mechanism not only ensures high local concentrations of encapsulated analgesics but also simultaneously scavenges harmful radicals, offering a dual-action approach that maximizes therapeutic efficacy while minimizing the systemic toxicities associated with conventional pain management. For example, Zhang's group identified the calcium voltage-gated channel auxiliary *α*2*δ*1 subunit as a potential analgesic target by screening differentially expressed genes in the DRG of rats with peripheral nerve injury. They then developed a mimicking peptide (AD peptide) to relieve neuropathic pain and packaged it into a nanomicelle, which was assembled from an amphiphilic conjugate comprising polyethylene glycol, phenylboronic acid pinacol ester, and Tempol [208]. After intravenous injection into a rat model of nerve injury-induced neuropathic pain, the nanomicelle preferentially accumulated in the injured nerve. The ROS-responsive characteristic of the nanomicelle enabled the release of AD peptide and other components, which induced anti-inflammatory, anti-apoptotic, and anti-oxidative stress effects, promoting nerve regeneration and affording synergistic efficacy in treating nerve injury-induced neuropathic pain. By leveraging the intersection of materials engineering and pain pathophysiology, ROS-responsive NPs provide a sophisticated platform for on-demand drug delivery, effectively addressing the limitations of conventional systemic analgesics. In addition, through rational design and specific chemical modification, nanomaterials with inherent analgesic effects can also be endowed with ROS responsiveness, thereby achieving precise targeted therapy for pain. Despite their precision, ROS-responsive systems still face significant challenges regarding the heterogeneity of oxidative levels and the complexity of neural anatomy.

##### 4.2.1.3 Enzyme

**Fig. 12:**
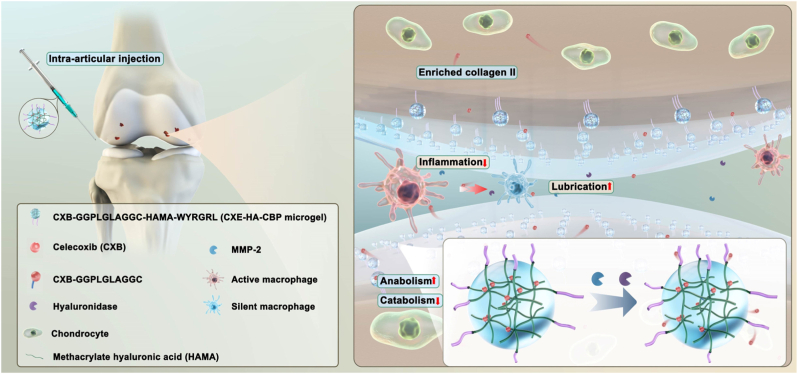
Schematic representation of celecoxib and collagen II binding peptide-modified hyaluronic acid-based microgel for treating OA *via* inflammation suppression, chondrocyte protection, and joint lubrication improvement. Reprinted with permission from Ref. [210]. Copyright Elsevier 2024.

Enzymes function as critical pathological mediators within chronic pain microenvironments. Specifically, the elevated expression of esterases and MMPs at inflammatory sites, and the activation of proteases following nerve injury, not only regulate pain-related signaling but also serve as endogenous triggers for precision drug delivery [209]. By incorporating enzyme-sensitive peptide crosslinkers, researchers can engineer hydrogels or NPs that degrade selectively at protease-rich sites. For instance, Chen's group presented a methacrylate hyaluronic acid (MAHA) microgel for the on-demand release of the COX-2 inhibitor celecoxib (CXB) (Fig. 12) [210]. In this system, CXB was conjugated to MAHA *via* an MMP-2-sensitive peptide linker (GGPLGLAGGC). Upon exposure to the MMP-2-rich environment of OA joints, the linker was cleaved, triggering the controlled release of CXB to suppress inflammation and protect chondrocytes. The microgel's dense crosslinking ensured slow enzymatic degradation and sustained drug release. Furthermore, a collagen II binding peptide on the microgel surface anchored the system to the cartilage, creating a lubricating protective coating. This dual-functional design enhanced joint retention and lubrication, prolonging therapeutic efficacy while minimizing injection frequency and side effects.

Furthermore, elevated enzyme levels can also serve as therapeutic targets. For example, Bunnett's group targeted protease-activated receptor 2 (PAR_2_), which mediated pain in inflammatory bowel disease (IBD) upon activation by intestinal proteases [211]. They engineered dendritic and core-shell NPs to deliver the negative allosteric modulator AZ3451 specifically to intracellular signaling compartments. Within the endosomal microenvironment, NPs-encapsulated AZ3451, unlike its unencapsulated counterpart, rapidly and completely inhibited the activation of PAR_2_, Gαq, and β-arrestin-1 in early endosomes, as well as ERK signaling in the cytosol and nucleus. This targeted strategy effectively attenuated both IBD-related inflammation and pain. Furthermore, Liu and colleagues developed mannose-coated superparamagnetic iron oxide NPs (mSPIONs) to alleviate vincristine-induced peripheral neuropathic pain [156]. These NPs facilitated active targeting *via* mannose receptor-mediated uptake by macrophages. Once internalized, the mSPIONs exhibited enzyme-mimetic antioxidant activity to scavenge ROS. This antioxidant functionality was further amplified in response to the elevated expression of pro-inflammatory enzymes, such as inducible nitric oxide synthase and p47phox. By downregulating these enzymes and suppressing ROS generation, the mSPIONs effectively alleviated neuropathic pain. Although promising, the primary shortages of enzyme-responsive drug delivery for pain relief stem from biological unpredictability, where inconsistent enzyme levels between patients lead to unreliable drug release or off-target side effects. Additionally, the high technical complexity of manufacturing these smart carriers makes them difficult to scale and prone to premature leakage. Consequently, while biologically innovative, they currently face significant hurdles in achieving the clinical stability and cost-effectiveness required for standard medical use.

#### 4.2.2 External stimulus-responsive strategy

In contrast to biological triggers, external stimuli offer an unparalleled level of spatiotemporal control by allowing clinicians to trigger drug release/activation through applied physical energy. These strategies involve the use of non-invasive external sources, such as ultrasound, light, magnetic fields, to activate command-responsive nanomedicine at the precise moment pain relief is required. By decoupling the delivery/activation mechanism from the body's unpredictable internal chemistry, external stimuli provide a predictable and tunable approach to pain management.

##### 4.2.2.1 Light

Light is an increasingly popular tool in clinical and experimental medicine because it allows for spatiotemporal control. The applications of light for pain relief generally fall into three categories: direct therapy, light-activated drug delivery, and advanced nanomaterial-based modulation. Firstly, photobiomodulation (PBMT), formerly known as low-level laser therapy, is the most common direct application [212,213]. It doesn't rely on heat, but rather on the biological response of cells to specific wavelengths (usually red or near-infrared). In PBMT, photons trigger intracellular chemical changes by activating mitochondrial cytochrome *c* oxidase and other key chromophores. These photoreceptors initiate a cascade of neuroprotective responses that enhance cellular metabolism, stimulate blood flow, and promote neurogenesis. Furthermore, this process effectively mitigates inflammation and oxidative stress, facilitating tissue repair and pain relief. For instance, Sandoz's group demonstrated that 365 nm illumination activated TRAAK, a two-pore domain potassium channel involved in pain inhibition [214]. This activation was driven by the oxidation of a native methionine at the TRAAK regulatory fenestration site, triggering a conformational shift from an inactive (down) to an active (up) state. In rodent models, non-invasive skin exposure to 365 nm light was sufficient to activate endogenous TRAAK and silence nociceptors. This produced potent, long-lasting analgesia that outperformed standard treatments in two neuropathic pain models.

**Fig. 13:**
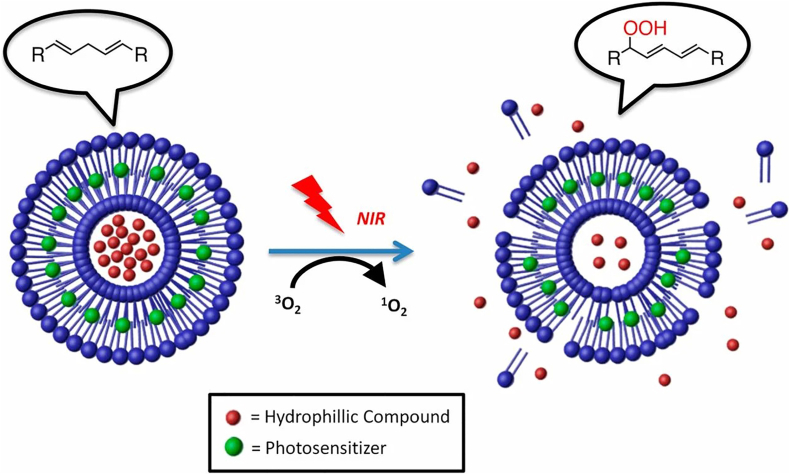
Schematic of photo-triggered cargo release from liposomes.

Secondly, light-activated drug delivery provides a robust solution to the challenges of rapid drug clearance and transient efficacy, effectively reducing dosing frequency while improving analgesic outcomes. For instance, Li and colleagues engineered a nanoplatform utilizing a mesoporous polydopamine photothermal core integrated with phase-change gating materials to create an near-infrared (NIR)-responsive switch [215]. This design enabled the release of anti-inflammatory agents to be precisely synchronized with irradiation duration, facilitating a therapeutic modality characterized by exceptional spatiotemporal control. Furthermore, because interrupting peripheral nerve conduction is a potent analgesic route, local anesthetics can be encapsulated in light-responsive nanocarriers for on-demand release. These systems remain inert until triggered by exogenous optical stimulation, at which point they induce a neural blockade. Rwei et al. demonstrated this using photosensitive liposomes loaded with tetrodotoxin (TTX) and a NIR photosensitizer [216]. Upon 730 nm NIR irradiation, the photosensitizer generated singlet oxygen to induce membrane peroxidation and TTX release (Fig. 13). In rat sciatic nerve models, this platform provided repeatable, inducible blockades with intensity and duration tuned *via* irradiation parameters.

Finally, the integration of light with advanced nanomaterial-based modulation has emerged as a sophisticated frontier. A primary example is the use of NIR-responsive nanomaterials in photothermal therapy, a potent strategy for managing chronic OA pain. As previously introduced, by using specific nanomedicine, NIR-triggered localized heating selectively activated TRPV1 receptors, ultimately restoring locomotor function, providing significant analgesia, and slowing cartilage degeneration [141]. Overall, light-responsive NPs provide a noninvasive, repeatable, and tunable framework for on-demand analgesia. By leveraging multiple synergistic mechanisms, these nanotherapeutic platforms represent a versatile frontier in chronic pain management. However, significant challenges, including limited light penetration, complex biological barriers, and long-term toxicity, continue to impede the clinical translation of these light-responsive systems.

##### 4.2.2.2 Ultrasound

Due to its noninvasive nature, superior tissue penetration, and capacity for precise spatiotemporal control, ultrasound has emerged as a prominent external trigger for nanotherapeutic pain management [217,218]. The analgesic mechanisms of this approach are generally divided into two primary modalities. First, ultrasound-mediated triggers can facilitate the controlled release of local anesthetics, achieving highly targeted neural blockade at the site of pain. For instance, Qi et al. developed glycosylated chitosan-coated mesoporous organosilica NPs loaded with ropivacaine [219]. These ultrasound-activated carriers enable on-demand analgesic release, demonstrating potent *in vivo* pain relief alongside excellent histocompatibility. Similarly, Luan's group engineered an ultrasound-responsive nanoplatform utilizing dendritic mesoporous silica co-loaded with levobupivacaine and perfluoropentane (PFP) [220]. The vaporization of liquid PFP was triggered by ultrasound irradiation to produce a gas environment. Subsequently, the enhanced cavitation effect could improve the release of levobupivacaine to achieve pain relief under a moderate-intensity ultrasound irradiation. This design ensured the system remain stable and drug-retentive until ultrasound triggered a phase change, facilitating rapid, repeatable, and controllable nerve blockade in the perineural region.

**Fig. 14:**
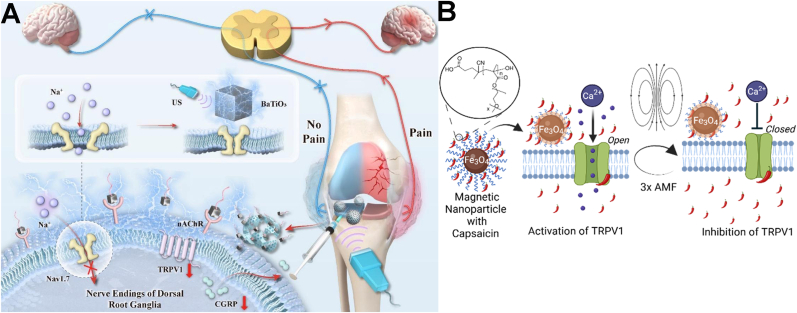
(A) Schematic diagram of analgesic microspheres alleviating OA pain by ultrasound mediated piezoelectric effect. Reprinted with permission from Ref. [221]. Copyright Elsevier 2025. (B) Schematic diagram of: POEGMA-coated iron oxide NPs as alternating magnetic-field-triggered capsaicin reservoirs for on-demand chronic pain management. Reprinted with permission from Ref. [222]. Copyright America Chemical Society 2025.

Furthermore, ultrasound can activate piezoelectric nanomaterials to produce local electrical stimulation. These generated fields directly influence the activity of ion channels or receptors involved in pain signaling, offering a non-invasive neuromodulation strategy. For instance, in the context of OA, Li et al. engineered the ultrasound-responsive piezoelectric microsphere BaTiO_3_-RVG@GA/HA (Fig. 14A) [221]. Upon activation, these microspheres generated electrical pulses at DRG terminals, inducing slow inactivation of the Na_V_1.7. By suppressing Na ^+^ influx, this system effectively interrupted nociceptive signaling to alleviate pain. Similarly, as previously noted, Bu's group developed PVP-modified BiOIO_3_ piezoelectric nanosheets that regulated TRPV1 in response to ultrasound, specifically for treating cancer-induced bone pain [143]. In summary, ultrasound-triggered analgesia has shifted from simple on-demand drug release toward sophisticated piezoelectric modulation of molecular pain targets. This evolution has established ultrasound-responsive nanotherapeutics as a cornerstone of contemporary chronic pain research.

##### 4.2.2.3 Magnetic fields

Harnessed for their unique responsiveness to external magnetic fields, magnetic NPs (MNPs) enable the remote and noninvasive management of chronic pain. Their capacity for spatially controllable modulation makes them ideal candidates for addressing the complex requirements of inflammatory and neuropathic pain models, which necessitate targeted, long-term therapeutic delivery [223]. Early research utilized MNPs primarily as addressable carriers for analgesic and anti-inflammatory agents. By leveraging external magnetic fields, these systems enhanced the localized accumulation and retention of therapeutic payloads, maximizing efficacy while mitigating systemic toxicity. For instance, Wu et al. covalently conjugated ketorolac to superparamagnetic iron oxide NPs [224]. Following intrathecal administration, magnetic field polarity was used to precisely localize the drug within the spinal cord. This targeted enrichment significantly enhanced analgesia in inflammatory pain models *via* COX inhibition, establishing a noninvasive paradigm for localized chronic pain therapy. Notably, accumulating evidence indicates that MNPs can directly modulate pain pathways independent of their role as drug carriers. For example, Lu's group demonstrated that amino-functionalized ultrasmall superparamagnetic iron oxide NPs (USPIO-101) exerted intrinsic spinal analgesia by suppressing long-term potentiation in the dorsal horn [225]. This finding redefined MNPs as active regulators of pain signaling rather than passive delivery vehicles.

Furthermore, integrating MNPs with alternating magnetic fields (AMFs) enables localized hyperthermia to wirelessly modulate pain-related ion channels. For example, Romero's group utilized iron oxide MNPs coated with thermoresponsive poly(oligo (ethylene glycol) methyl ether methacrylate) (POEGMA), which served as a capsaicin reservoir (Fig. 14B) [222]. Under AMF exposure, the MNPs dissipated heat, triggering a conformational change in the POEGMA coating to release the encapsulated capsaicin. This single-dose release activated TRPV1 receptors in over 75% of hippocampal neurons, a mechanism that typically correlates with increased pain sensitivity. This strategy offers a wireless, repeatable alternative to implanted electrodes, significantly broadening the therapeutic potential of magnetic nanomaterials in pain management. Consequently, magnetic NPs-based therapy has evolved from simple field-assisted delivery into a multifaceted platform combining spatial targeting, controlled release, and direct molecular modulation for precision pain management.

## 5 Conclusion and future perspectives

Effective pain management remains a paramount medical concern. This review provides a comprehensive overview of recent breakthroughs in nanomedicine tailored for pain management. While nanomaterials have shown promise in pain relief, their translation into clinical practice remains hindered by a variety of unresolved problems and challenges.

1. Currently, most nanomaterials for pain relief necessitate intrathecal injection, with few exceptions possessing sufficient BBB penetrating efficiency to permit intravenous administration. Consequently, there is a critical need for nanoplatforms adaptable to diverse clinical scenarios, including subcutaneous, local lesion, and perineural injections. While intravenous injection is technically simple and ideal for systemic or multifocal pain, intrathecal delivery offers rapid onset and high spinal concentration by bypassing the BBB. For long-term maintenance, subcutaneous injection provides a sustained-release alternative. Conversely, local lesion injection ensures precise deposition at pathological sites, minimizing systemic exposure. Finally, perineural targeted injection specifically blocks peripheral pain signals, offering high specificity for neuropathic conditions. Despite these advantages, all such routes remain inherently invasive, presenting a persistent barrier to patient compliance.

Therefore, there is also a demand for analgesic nanomaterials compatible with minimally invasive delivery strategies, such as oral, intranasal, and microneedle-based transdermal routes. Oral administration offers unparalleled convenience for chronic pain management, while the intranasal route provides a direct nose-to-brain pathway that circumvents the BBB for rapid central analgesia. Similarly, microneedle systems serve as a painless alternative to hypodermic needles, facilitating sustained transdermal permeation while avoiding hepatic first-pass metabolism. The rational design and optimization of nanocarriers tailored to these modalities are essential to surmount the drawbacks of conventional regimens and accelerate the clinical translation of chronic pain nanotherapeutics.

2. While bypassing the BBB through alternative routes, such as intranasal and intrathecal injection, is a promising strategy, developing intravenous nanomedicines capable of efficient BBB penetration remains a formidable necessity for treating severe central neuropathic pain. The highly restrictive tight junctions of the BBB sequester the vast majority of systemic therapeutics from the central nervous system. To overcome this, future nanomedicines can be engineered with active targeting ligands, such as transferrin or monoclonal antibodies, to exploit receptor-mediated transcytosis. Furthermore, the synergy between nanomaterials and localized external stimuli, such as low-intensity focused ultrasound, represents a critical frontier for transiently permeabilizing the BBB to achieve therapeutic concentrations.
3. The clinical translation of nanomaterial-based analgesics is severely hindered by biosafety and toxicity concerns, most notably unexpected immune activation, inflammatory responses, and long-term tissue damage. Following administration, nanocarriers are often cleared by the reticuloendothelial system, potentially triggering immune rejection or histological lesions in metabolic organs. Furthermore, the absence of standardized safety guidelines tailored to the nanoscale makes it difficult to characterize complex biological behaviors, such as cumulative toxicity and reproductive risks. Because the mechanisms governing the long-term metabolism of these nanoplatforms remain unclear, their clinical application in chronic pain therapy remains restricted. To date, only a few lipid or polymeric carriers have entered clinical practice, and none has yet been approved specifically for chronic pain. This gap underscores the urgent need for unified evaluation standards and biocompatible design optimizations to facilitate clinical transition. Prioritizing biodegradable and biomimetic nanoplatforms is essential for improving the safety profiles and therapeutic precision of future nanomedicines.
4. The synthesis of nanomedicines requires meticulous control over critical parameters, including particle size, surface charge, and distribution. As these factors dictate targeting efficiency, biocompatibility, and efficacy, even minor deviations can compromise product quality. In industrial settings, environmental variables and the limitations of scalable manufacturing technologies often hinder consistent batch production. Furthermore, many nanoplatforms require complicated synthetic procedures and harsh reaction conditions that are difficult to replicate at scale. These technical barriers, coupled with the high costs of specialized production, undermine the economic viability and regulatory approval of nanomedicines, ultimately stalling their transition from laboratory to clinic. Furthermore, ensuring sterility and long-term storage stability without compromising the structural integrity of the nanocarriers adds another layer of difficulty.
5. The majority of existing studies rely on conventional mouse experiments, but these may not accurately predict therapeutic efficacy in humans. In particular, most pain models established in mice differ substantially from real clinical pain conditions in humans, with highly variable and heterogeneous underlying etiological factors between the two species. Additionally, inherent interspecies discrepancies in disease progression, pathological manifestations and immune response further limit the translational value of mouse findings. Furthermore, rodent pain evaluation heavily relies on evoked reflexive behaviors (e.g., paw withdrawal thresholds), which poorly reflect the spontaneous, emotional, and highly subjective nature of human chronic pain. To ensure reliable clinical outcomes, it is imperative to validate these nanomedicines in more advanced platforms.
6. Chronic pain is an extremely subjective and highly heterogeneous disorder, with therapeutic efficacy varying substantially across individuals due to distinct genetic backgrounds, biological sex, disease progression stages, and unique tissue microenvironmental characteristics. In this context, the conventional one-size-fits-all therapeutic strategy for chronic pain has proven increasingly insufficient to meet clinical demands. Therefore, the development of personalized nanomedicines has emerged as a critical research direction for future pain management. This strategy encompasses the rational design of theranostic nanomaterials that integrate diagnostic imaging and targeted drug delivery, enabling clinicians to dynamically monitor *in vivo* drug accumulation at lesion sites as well as real-time alterations of localized biochemical parameters, such as ROS levels and pH microenvironment. Furthermore, engineering high-sensitivity and multi-stimuli-responsive smart nanocarriers that can autonomously modulate drug release kinetics in response to the severity of local inflammatory markers will drive a transformative advancement toward precise and individualized chronic pain therapy.

Resolving these outstanding issues will significantly expand the applications of nanomaterials in pain management. By leveraging the unique properties of nanomaterials, researchers have achieved unprecedented precision in targeting the biological origins of pain, thereby minimizing systemic dosages while bolstering long-term therapeutic efficacy and safety. The integration of nanotechnology to both identify and modulate novel pain-related molecular targets will represent a burgeoning frontier in the quest for personalized pain relief.

**Table:** Abbreviations

Abbreviations	Full Term
AAV	Adeno-associated virus
ADAM8	A disintegrin and metalloproteinase protein 8
AMFs	Alternating magnetic fields
BIONSs	BiOIO_3_ nanosheets
cAMP	Cyclic adenosine monophosphate
CAT	Catalase
CBP	Collagen II binding peptide
CeO_2_	Cerium oxide nanoparticle
CFA	Complete Freund's adjuvant
CIBP	Cancer-induced bone pain
CINP	Chemotherapy-induced neuropathic pain
CNS	Central nervous system
COX	Cyclooxygenase
CPSP	Central post-stroke pain
CRISPR-Cas9	Clustered regularly interspaced short palindromic repeats-Cas9
CUB	Ce-UiO-66
CXB	Celecoxib
DRG	Dorsal root ganglion
EVs	Extracellular vesicles
FST	Follistatin
GPx	Glutathione peroxidase
HA	Hyaluronic acid
MAHA	Methacrylate hyaluronic acid
HSV-1	Herpes simplex virus type 1
IBD	Inflammatory bowel disease
LA	Local anesthetics
LNPs	Lipid NPs
mAbs	Monoclonal antibodies
MgO	Magnesium oxide
MMPs	Matrix metalloproteinases
MNPs	Magnetic NPs
MOFs	Metal-Organic Frameworks
mRNA	Messenger RNA
MSCs	Mesenchymal stem cells
MSNs	Mesoporous silica NPs
mSPIONs	Mannose-coated superparamagnetic iron oxide NPs
NGF	Nerve growth factor
NIR	Near-infrared
NK1R	Neurokinin 1 receptor
NLCs	Nanostructured lipid carriers
NMDA	N-methyl-d-aspartate
NPs	Nanoparticles
NSAIDs	Non-steroidal anti-inflammatory drugs
NSCs	Neural stem cells
NSTX	Neosaxitoxin
nZnO	Nano ZnO
OA	Osteoarthritis
PAMAM	Poly(amidoamine)
PAR_2_	Protease-activated receptor 2
PBMT	Photobiomodulation
PCL	Poly(ε-caprolactone)
p-DBSN	P-dodecylbenzene sulfonamide
PFP	Perfluoropentane
PLA	Poly(lactic acid)
PLGA	Poly(lactic-co-glycolic acid)
PLL	Poly(L-lysine)
POEGMA	Poly(oligo (ethylene glycol) methyl ether methacrylate)
PS	Phosphatidylserine
RA	Rheumatoid arthritis
Ropi	Ropivacaine
ROS	Reactive oxygen species
Ru	Ruthenium
siRNA	Small interfering RNA
SLNs	Solid lipid NPs
SNI	Spared nerve injury
SOD	Superoxide dismutase
SP	Substance P
TF	Transferrin
TRPA1	Transient receptor potential A1
TRPV1	Transient receptor potential vanilloid 1
TTX	Tetrodotoxin
USPIO-101	Ultrasmall superparamagnetic iron oxide NPs
WHO	World Health Organization
ZFP	Zinc finger protein
ZIF	Zeolitic imidazolate framework
ZnO	Zinc oxide

## CRediT authorship contribution statement

**Zihan Xue:** Writing – original draft, Writing – review & editing. **Chengfeng Zhang:** Writing – original draft. **Jingyi Wang:** Writing – original draft. **Jianing Li:** Writing – original draft. **Faming Wang:** Funding acquisition, Supervision, Writing – review & editing. **Zhongping Chen:** Supervision, Writing – review & editing. **Yan Zhang:** Funding acquisition, Supervision, Writing – original draft, Writing – review & editing.

## Declaration of competing interest

The authors declare that they have no known competing financial interests or personal relationships that could have appeared to influence the work reported in this paper.

## Data Availability

No data was used for the research described in the article.
